# YAP1 enhances NF-κB-dependent and independent effects on clock-mediated unfolded protein responses and autophagy in sarcoma

**DOI:** 10.1038/s41419-018-1142-4

**Published:** 2018-10-31

**Authors:** Adrian Rivera-Reyes, Shuai Ye, Gloria E. Marino, Shaun Egolf, Gabrielle E. Ciotti, Susan Chor, Ying Liu, Jessica M. Posimo, Paul M. C. Park, Koreana Pak, Yael Babichev, Jaimarie Sostre-Colón, Feven Tameire, Nektaria Maria Leli, Constantinos Koumenis, Donita C. Brady, Anthony Mancuso, Kristy Weber, Rebecca Gladdy, Jun Qi, T. S. Karin Eisinger-Mathason

**Affiliations:** 1 Abramson Family Cancer Research Institute, Department of Pathology & Laboratory Medicine, Penn Sarcoma Program, University of Pennsylvania Perelman School of Medicine, Philadelphia, PA USA; 2 Department of Cancer Biology, University of Pennsylvania Perelman School of Medicine, Philadelphia, PA USA; 3 Department of Medicine, Harvard Medical School, Boston, MA 02115 USA; 4 Department of Surgery and Institute of Medical Science, University of Toronto, Toronto, ON Canada; 5 Lunenfeld-Tanenbaum Research Institute, Sinai Health System, Toronto, ON Canada; 6 Department of Radiation Oncology, University of Pennsylvania Perelman School of Medicine, Philadelphia, PA USA; 7 Department of Radiology, University of Pennsylvania Perelman School of Medicine, Philadelphia, PA USA; 8 Department of Orthopedic Surgery, Penn Sarcoma Program, University of Pennsylvania Perelman School of Medicine, Philadelphia, PA USA

## Abstract

Terminal differentiation opposes proliferation in the vast majority of tissue types. As a result, loss of lineage differentiation is a hallmark of aggressive cancers, including soft tissue sarcomas (STS). Consistent with these observations, undifferentiated pleomorphic sarcoma (UPS), an STS subtype devoid of lineage markers, is among the most lethal sarcomas in adults. Though tissue-specific features are lost in these mesenchymal tumors they are most commonly diagnosed in skeletal muscle, and are thought to develop from transformed muscle progenitor cells. We have found that a combination of HDAC (Vorinostat) and BET bromodomain (JQ1) inhibition partially restores differentiation to skeletal muscle UPS cells and tissues, enforcing a myoblast-like identity. Importantly, differentiation is partially contingent upon downregulation of the Hippo pathway transcriptional effector Yes-associated protein 1 (YAP1) and nuclear factor (NF)-κB. Previously, we observed that Vorinostat/JQ1 inactivates YAP1 and restores oscillation of NF-κB in differentiating myoblasts. These effects correlate with reduced tumorigenesis, and enhanced differentiation. However, the mechanisms by which the Hippo/NF-κB axis impact differentiation remained unknown. Here, we report that YAP1 and NF-κB activity suppress circadian clock function, inhibiting differentiation and promoting proliferation. In most tissues, clock activation is antagonized by the unfolded protein response (UPR). However, skeletal muscle differentiation requires both Clock and UPR activity, suggesting the molecular link between them is unique in muscle. In skeletal muscle-derived UPS, we observed that YAP1 suppresses PERK and ATF6-mediated UPR target expression as well as clock genes. These pathways govern metabolic processes, including autophagy, and their disruption shifts metabolism toward cancer cell-associated glycolysis and hyper-proliferation. Treatment with Vorinostat/JQ1 inhibited glycolysis/MTOR signaling, activated the clock, and upregulated the UPR and autophagy via inhibition of YAP1/NF-κB. These findings support the use of epigenetic modulators to treat human UPS. In addition, we identify specific autophagy, UPR, and muscle differentiation-associated genes as potential biomarkers of treatment efficacy and differentiation.

## Introduction

Soft tissue sarcomas (STS) are a complex set of tumors that arise in mesenchymal tissues, including muscle, fat, cartilage, and connective tissue. Owing to their karyotype complexity, variety of subtypes, and the lack of known drivers, adult sarcomas are very poorly understood. Treatment options are generally limited to radiation and surgery, as inadequate characterization has precluded the development of targeted therapies^1–3^. Our current work focuses on undifferentiated pleomorphic sarcoma (UPS), an aggressive adult tumor found in skeletal muscle. Muscle-derived UPS is a commonly diagnosed subtype relative to other sarcomas and is particularly difficult to treat^4^. We found that the central Hippo effector, Yes-associated protein 1 (YAP1), is stabilized in human UPS tumors and promotes a pro-proliferation transcriptional program^5,6^. YAP1 is unusually stable in UPS and potentially other sarcomas due to epigenetic silencing of its inhibitor, Angiomotin (AMOT)^7^, and Hippo kinase copy number loss^5^. These perturbations stabilize YAP1 at the protein level; enhance its nuclear localization and subsequent transcriptional activity^8^. Though well-studied in epithelial tumors, the specific downstream effectors of YAP1 in sarcomas are not well characterized.

Skeletal muscle-derived UPS is thought to develop from muscle progenitor cells/satellite cells^9^, which undergo proliferation as immature myoblasts before differentiating into mature muscle fibers. YAP1 and NF-κB signaling are essential for myoblast proliferation and these pathways must be inhibited to permit terminal differentiation^10–14^. Thus, during normal muscle development inhibition of NF-κB and YAP1 are associated with loss of proliferative capacity, and upregulation of muscle differentiation markers like MYOD and MEF2C. Recently, we discovered that YAP1 controls NF-κB activity in muscle-derived UPS, by inhibiting expression of ubiquitin specific peptidase 31 (USP31) a negative regulator of NF-κB^7^. In the absence of a specific inhibitor for YAP1 we used a combination of the epigenetic modulators suberoylanilide hyroxamic acid (SAHA; Vorinostat), and the BET bromodomain inhibitor JQ1, which we recently discovered suppresses YAP1 activity. Though SAHA/JQ1 treatment has widespread effects, we use these tools to interrogate and then validate YAP1-mediated signaling and phenotypes. Importantly, SAHA/JQ1 treatment upregulated a transcriptional program associated with muscle differentiation in UPS cells. Here we report that inhibition of YAP1 and/or NF-κB recapitulates several key aspects of SAHA/JQ1-mediated differentiation.

Interestingly, we observed that NF-κB signaling oscillates over time in muscle precursor cells^7^ and other tissues^15,16^. Consistent with these findings, normal myoblast proliferation and muscle differentiation have been linked to peripheral circadian oscillation^17–19^. The circadian clock is a 24-hour molecular signaling hub that regulates proliferation via control of metabolic processes^20,21^ and is regulated by positive and negative feedback loops^22,23^. The main transcriptional components, CLOCK and BMAL1, form a heterodimer that binds to an E-box in the promoters of target genes, such as *PERIOD* (*PER*) and CRYPTOCHROME (*CRY*), enhancing their expression, which in turn inactivates CLOCK and BMAL1 heterodimers^23,24^. Shuttling of PER and CRY proteins from the cytoplasm to the nucleus regulates the feedback loop. Importantly, a growing body of literature suggests that disruption of circadian oscillation promotes tumorigenesis in a variety of cancer settings^25–28^.

Recent studies have shown that clock activity is linked to a number of pathways associated with muscle differentiation including the unfolded protein response (UPR)^29–31^ and autophagy^21,32–34^. Cross-talk between UPR and autophagy has also been observed but the molecular mechanisms and coordination of these processes have yet to be elucidated^35–38^. Importantly, YAP1 plays a role in cancer related autophagic flux^39^ and UPR^40^. Based on these observations we hypothesized that YAP1 and/or NF-κB helps control differentiation in muscle tissues and tumors via regulation of the circadian clock and downstream processes. Here we show that YAP1/NF-κB, control the switch between differentiation and proliferation in sarcoma by suppressing the circadian clock and UPR. Interestingly, we observed that YAP1 also suppresses autophagy but in an NF-κB-independent manner.

## Materials and methods

### Mouse models

#### Genetically Engineered Mouse Models (GEMM)

All experiments were performed in accordance with NIH guidelines and were approved by the University of Pennsylvania Institutional Animal Care and Use Committee. We generated *Kras*^*G12D+*^; *Trp53*^*fl/fl*^; *YAP1*^*fl/fl*^ (KPY) and *Kras*^*G12D+*^; *Trp53*^*fl/fl*^; *Rela*^*fl/fl*^ (KPR) mice by crossing KP with *YAP1*^*fl/fl*^ and *Rela*^*fl/fl*^ animals. Tumors were generated by injection of a calcium phosphate precipitate of adenovirus expressing Cre recombinase (University of Iowa) into the right gastrocnemius muscle of 3–6-month-old mice.

#### In vivo drug treatment

For in vivo drug studies, total 44 (*n* = 11 per group) autochthonous KP mice were randomly divided into four groups to receive different treatments once tumors reached 100 mm^3^, and injected for up to 20 days. The mice are killed 24 h after the tumor volume reaches 2000 mm^3^). (1) Vehicle group (10% Hydroxypropyl-β-cyclodextrin plus dimethyl sulfoxide (DMSO) was diluted daily in sterile 45% PEG/55% H_2_O); (2) JQ1 and SAHA combination treatment group (drug were diluted in respective vehicles). Treatment method for drug combination group: (1) 25mg/kg SAHA + 50mg/kg JQ1 for first 5 days. (2) 25 mg/kg SAHA+25 mg/kg JQ1 each other day for 10 days. (3) 25 mg/kg SAHA + 50 mg/kg JQ1 for 2 days. (4) Then mice with tumors received 25 mg/kg SAHA with 25 mg/kg JQ1 for 3 days. Mice without tumors received 5 mg/kg SAHA and 5 mg/kg JQ1 for 3 days). JQ1 was provided by Jun Qi (Dana-Farber Cancer Institute) and SAHA was purchased from Cayman Chemical. HP-β-CD and PEG400 were obtained from Sigma-Aldrich.

#### Oncomine and TCGA survival analysis

We used the publically available database Detwiller et al. via the Oncomine Research Premium edition software (version 4.5, life Technologies) to query *PER1, PER2, CRY1, CRY2, ARNTL, TXNIP, DDIT3, FASN, CPT1A, CPT1B* gene expression in MFH/UPS. We also evaluated human patient survival using the TCGA sarcoma data set. Kaplan–Meier analyses were performed for overall survival of patients.

### Cell lines

KP230, KP250, and KIA cell lines were derived from UPS mouse tumors as described in^41^. Human HT-1080, HEK-293T cell lines were purchased from ATCC (Manassas, VA, USA). STS-109 cell line was derived from human UPS patients. STR analysis was performed at the time of derivation and confirmed in April 2015. Cells were purchased, thawed, and then expanded in the laboratory. Multiple aliquots were frozen down within 10 days of initial resuscitation. For experimental use, aliquots were resuscitated and cultured for up to 20 passages (4–6 weeks) before being discarded. Cells were cultured in Dulbecco's Modified Eagle's medium (DMEM) with 10% (vol/vol) fetal bovine serum (FBS), 1% penicillin/streptomycin, 1% glutamine, at 5% CO_2_ and 37°C. All cell lines were confirmed to be negative for mycoplasma contamination.

#### Drug treatments

Cells were treated with SAHA (2 µm) and JQ1 (0.5 µm) either individually or in combination for the time indicated in the figure legends. Drugs were refreshed for any cells treated for longer than 48 h.

#### Lentiviral transduction

shRNA-mediated knockdown of Per1: TRCN0000075403; Arntl: TRCN0000095055; Txnip: TRCN0000182360; Ddit3: TRCN0000103709; RelA: TRCN0000055344, TRCN0000055346; and Yap1: TRCN0000095864, TRCN0000095865 were obtained as glycerol stocks from Dharmacon. Scramble shRNA was obtained from Addgene. High-copy plasmid purification was conducted for each shRNA using Clontech Laboratories Inc. NucleoBond Exta Midi kit (740410.50) according to the NucleoBond ^®^ Xtra Plasmid Purification Maxi protocol. shRNA plasmids were packaged by using the third-generation lenti-vector system (VSV-G, p-MDLG, and pRSV-REV) and expressed in HEK-293T cells. Supernatant was collected at 24 and 48 h after transfection and subsequently concentrated by using 10-kDa Amicon Ultra-15 centrifugal filter units (Millipore). After 72 h of lentiviral infection, cells were selected with puromycin (1.5 µg/ml) for 24–48 h. shRNA infected cells were treated with SAHA (2 µm) and JQ1 (0.5 µm) for 48 h kept under puromycin-selecting conditions.

### ChIP-seq

For tumor samples resected from UPS patients at the Hospital of the University of Pennsylvania, ~ 100 mg of tissue was minced into 1–2 mm pieces and incubated in 1% formaldehyde for 15 min. Formaldehyde was quenched with glycine at 0.125 m. Fixed tissue was homogenized for 60 sec with a Tissue Tearor Homogenizer (Biospec) at 30,000 rounds per minute. Homogenized tissue was washed with ice-cold phosphate-buffered saline (PBS) with 1× HALT protease inhibitor. For cell line ChIP-RX, samples were fixed for 10 min in 1% formaldehyde quenched with glycine and washed with PBS as above. 5e6 S2 cells (Drosophila Melanogaster) were added to each sample of 2.5e7 for ChIP-RX normalization in downstream analysis.

#### Transient transfections

SMARTpool: ON-TARGETplus Yap1 siRNA (M-100439-01-0005), Cry2 siRNA (L-040486-00-0005), Per1 siRNA (L-040487-00-0005), and non-targeting siRNA were purchased from Dharmacon.

#### UPR reporter assays

KP cells were plated in a six-well plate and transiently transfected using the Lipofectamine^TM^3000 protocol. Cells were transfected using the Addgene plasmids ATF4 5: 5’ATF4:GFP (#21852) and pEGFP-ATF6 (#32955). Fluorescence images were taken using Olympus IX2-UCB microscope, SensiCam^QE^ High Perfomance camera, and X-Cite^®^ Series 120PC. Images were taken using the Slidebook 6 program.

#### Immunoblots

Protein lysate was prepared in SDS/Tris (pH7.6) lysis buffer, separated by electrophoresis in 8–10% sodium dodecyl sulphate-polyacrylamide gel electrophoresis gels, transferred to nitrocellulose membrane, blocked in 5% non-fat dry milk, and probed with the following antibodies: rabbit anti-PER1 (ab3443; 1:250), rabbit anti-CRY2 (ab38872, 1:500) (Abcam), rabbit anti-BMAL1 (14020S; 1:1000), rabbit anti-YAP1 (4912; 1:1000), rabbit anti-GAPDH (2118; 1:1000), (Cell Signaling Technology), rabbit anti-CPT1A (15184-1-AP; 1:1000) (Proteintech), rabbi anti-TXNIP (ab188865, 1:1000) (Abcam), rabbit anti-CHOP (60304-1, Ig, 1:1000) (Proteintech), rabbit anti-FASN (3081, 1:1000) (Cell Signaling Technology), anti-LC3A/B (12741, 1:1000) (Cell Signaling Technology), rabbit anti-Caspase-3 (9662; 1:1000).

#### qRT-PCR

Total RNA was isolated from tissues and cells using the TRIzol regent (Life Technologies) and RNeasy Mini Kit (Qiagen). Reverse transcription of mRNA was performed using the High-Capacity RNA-to-cDNA Kit (Life Technologies). qRT-PCR was performed by using a ViiA7 apparatus. All probes were obtained from TaqMan “best coverage" (Life Technologies). Hypoxanthine phosphoribosyltransferase and or/succinate dehydrogenase subunit A (SDHA) was used as an endogenous control.

#### Luciferase assay

Plasmid pABpuro-BluF (46824; Addgene) was transfected into 293T cells (ATCC) to generate lentiviral particles in the supernatant. Viral supernatant was harvested and then concentrated by centrifugal filter units (Amicon Ultra-15, Millipore). Then Bmal reporter virus was transduced into KP230 cells. Positive Bmall reporter cells were selected by puromycin. For shRNA assays the Bmal reporter cell line was transduced with lentivirus expressing control or Yap1 shRNA. For drug studies, the Bmal reporter cells were treated with SAHA (2 µm)/JQ1 (0.5 µm) on time course. Luciferase activity was assayed using the Dual Luciferase Assay System (E2920, Promega) according to the manufacturer’s protocol on a Luminometer (GLOMAX, Promega). Results were calculated as fold induction.

#### Microarray and gene set enrichment analysis

Microarray services were provided by the UPENN Molecular Profiling Facility, including quality control tests of the total RNA samples by Agilent Bioanalyzer and Nanodrop spectrophotometry. All protocols were conducted as described in the Affymetrix WT Plus Reagent Kit Manual and the Affymetrix GeneChip Expression Analysis Technical Manual. In brief, 250 ng of total RNA was converted to first-strand cDNA using reverse transcriptase primed by poly(T) and random oligomers that incorporated the T7 promoter sequence. Second-strand cDNA synthesis was followed by in vitro transcription with T7 RNA polymerase for linear amplification of each transcript, and the resulting complementary RNA was converted to complementary DNA (cDNA), fragmented, assessed by Bioanalyzer, and biotinylated by terminal transferase end labeling. Five and a half micrograms of labeled cDNA were added to Affymetrix hybridization cocktails, heated at 99ºC for 5 min and hybridized for 16 h at 45ºC to Mouse Transcriptome 1.0 ST GeneChips (Affymetrix Inc., Santa Clara CA) using the GeneChip Hybridization oven 645. The microarrays were then washed at low (6 × sodium chloride sodium phosphate-EDTA buffer) and high (100mM MES, 0.1M NaCl) stringency and stained with streptavidin–phycoerythrin. Fluorescence was amplified by adding biotinylated anti-streptavidin and an additional aliquot of streptavidin–phycoerythrin stain. A GeneChip 3000 7G scanner was used to collect fluorescence signal. Affymetrix Command Console and Expression Console were used to quantitate expression levels for targeted genes; default values provided by Affymetrix were applied to all analysis parameters. Affymetrix cel (probe intensity) files were normalized and summarized using RMA-SST to the gene level using Expression Console software (v1.4.1). Inter sample variation was visualized using Principal Components Analysis in Partek Genomics Suite (v6.6, Partek, Inc., St. Louis, MO). Differential gene expression was tested using Significance Analysis of Microarrays (samr v2.0), yielding fold change, *q* value (false discovery rate) and d-score for each gene. We observed a small number of genes meeting our cutoffs for differential expression and so proceeded to gene set enrichment analysis (GSEA). Log_2_-transformed RMA-sst expression values were used as input to GSEA^42^ where enrichment was tested against the hallmark gene sets from the Molecular Signatures Database (MSigDB, v5.1, http://software.broadinstitute.org/gsea/msigdb/index.jsp)

#### Accession codes

Sequencing data reported in this paper have been deposited in NCBI's Gene Expression Omnibus and are accessible through GEO Series accession numbers GSE109920 and GSE109923.

#### C2C12 growth and differentiation

C2C12 murine myoblast cells were obtained from ATCC (Manassas, VA, USA). The cells grow as undifferentiated myoblasts in growth medium (20% FBS with 1% penicillin/streptomycin), and were passaged every 2–3 days at 50% sub-confluence. To induce differentiation cells were grown overnight to ~ 80% confluence in growth medium, and then switched to DMEM supplemented with 2% horse serum. Differentiation media was refreshed every 2 days.

#### Immunohistochemistry

Human UPS paraffin-embedded tissues were obtained from the Surgical Pathology group at University of Pennsylvania and stained thioredoxin-interacting protein (TXNIP) and C/EBP homologous protein (CHOP). Murine tumors from the KP GEMM and KP allografts were also sectioned and stained. IHC was performed on 5-micron tissue sections according to standard protocols. Sections were deparaffinized, rehydrated, and subjected to epitope retrieval and stained with The following antibody concentrations were used: rabbit anti-TXNIP (ab188865; 1:100) (Abcam), mouse anti-CHOP (Proteintech) (60304-1-IG; 1:250), rabbit anti-LC3B (Novus Biologicals; NB100-2220, 1:1000), rabbit anti-p62 (MBL International, PM045, 1:2000), mouse anti- Gadd34 (Novus Biologicals; NBP2-01787, 1:100) followed by peroxidase-based detection and counterstaining with haematoxylin using the Leica Bond Rx^m^ system with conditions described previously^43^. Representative photographs were taken on a Leica DMI6000B inverted light and fluorescent microscope with a 40 × oil objective. Images were blinded and positive staining was assessed in Image J. The color deconvolution macro was applied to images resulting in a 3,3′-diaminobenzidine staining (Color_2) generated window^44^. Using the threshold function, the total area of the tumor in pixels was recorded utilizing the same parameters for each tumor image. Areas staining positive by these parameters were selected and the positive-staining area in pixels was recorded. The positive-staining area of the tumor in pixels was divided by the total area of the tumor in pixels to determine the percentage positive-staining area.

#### Gas chromatography/mass spectrometry

After growth overnight, cultures were treated with either DMSO or 2 μm SAHA/0.5 μm JQ1. The cultures were allowed to grow for 48 h. Subsequently, the medium was changed to DMEM that contained 7 mM glucose, 4 mM glutamine, 75 μm [U-^13^C_16_]palmitic acid and 75 μm [U-^13^C_18_]oleic acid plus either DMSO or S/J. Both fatty acids were bound to fatty acid free albumin (two moles fatty acid per mole of albumin) before they were added to the medium. After 12 h of growth in the ^13^C-labeled medium, the cultures were harvested by cold methanol extraction. The cold methanol (80:20 methanol:water) was pre-cooled to − 80°C and added rapidly (2 ml/dish) after removal of the extracellular medium to prevent loss of intracellular metabolites. The cultures were stored at − 80°C prior to gas chromatography/mass spectrometry analysis (GC/MS) analysis. The methanol/cell mixtures were sonicated for 60 sec with a probe sonicator to disrupt all cell membranes and then centrifuged at 13,000 × g for 10 min. The supernatants were removed and transferred to sealable 4-ml glass tubes. Methanol and water were removed from the cell extracts with a heated (45°C) nitrogen evaporator. For GC/MS, the extracts were first derivatized with N,O-Bis(trimethylsilyl) trifluoroacetamide (BSTFA). The extracts in the 4-ml glass tubes were dissolved in 60 μl of pyridine. Subsequently, 60 μl BSTFA with 1% trimethylchlorosilane (Sigma-Aldrich, St. Louis, MO) were added and the mixtures were heated to 55C for 60 min. After cooling, the reaction mixtures were centrifuged at 13,000 × g for 5 min. The supernatant was transferred to 1.5 ml capped injection vials that were fitted with volume reducing glass sleeves. The derivatized samples were analyzed with an Agilent 7890A/5975C GC/MS system. Mass fragments were generated by electron impact at 70 eV. Helium was used as the carrier gas for the GC DB-5 column (30 m, with 10 m empty pre-column) at a flow rate of 1 ml/min. The injector was operated in splitless mode at 250°C. The column temperature profile was 0–3 min: 100°C, 3–17 min: ramp 10°C/min, 17–47 min: 240°C, 47–52 min: 300°C. The citrate retention time was 24.1 min. The mass scan range was 50–550 daltons. The relative enrichment of metabolites was calculated using IsoCor^45^.

#### Statistical analysis

Statistical analysis was performed using Prism (Graph Pad Software). Data are shown as mean ± standard error of mean or deviation. Data were reported as biological replicates. Experiments were performed in triplicate. Student *t* tests (unpaired two tailed) were performed to determine whether a difference between two values is statistically significantly different, with a *P* value < 0.05 considered significant.

## Results

### Clock gene expression is high in differentiating muscle and lost in UPS

We investigated the role of YAP1 using the *LSL*-*Kras*^G12D/+^; *Trp53*^fl/fl^ (KP) model of UPS. Adenovirus expressing Cre recombinase injected into the gastrocnemius muscle activates oncogenic Kras expression and deletes p53 in muscle progenitor cells, resulting in tumors that recapitulate human UPS^46,47^. Though *Kras* mutation is rare in human sarcomas, hyperactivation of the MAPK pathway downstream of activated KRAS is common in UPS and is an excellent prognostic indicator^48^. Importantly, Yap1 is stabilized in KP tumors^7^.

**Fig. 1:**
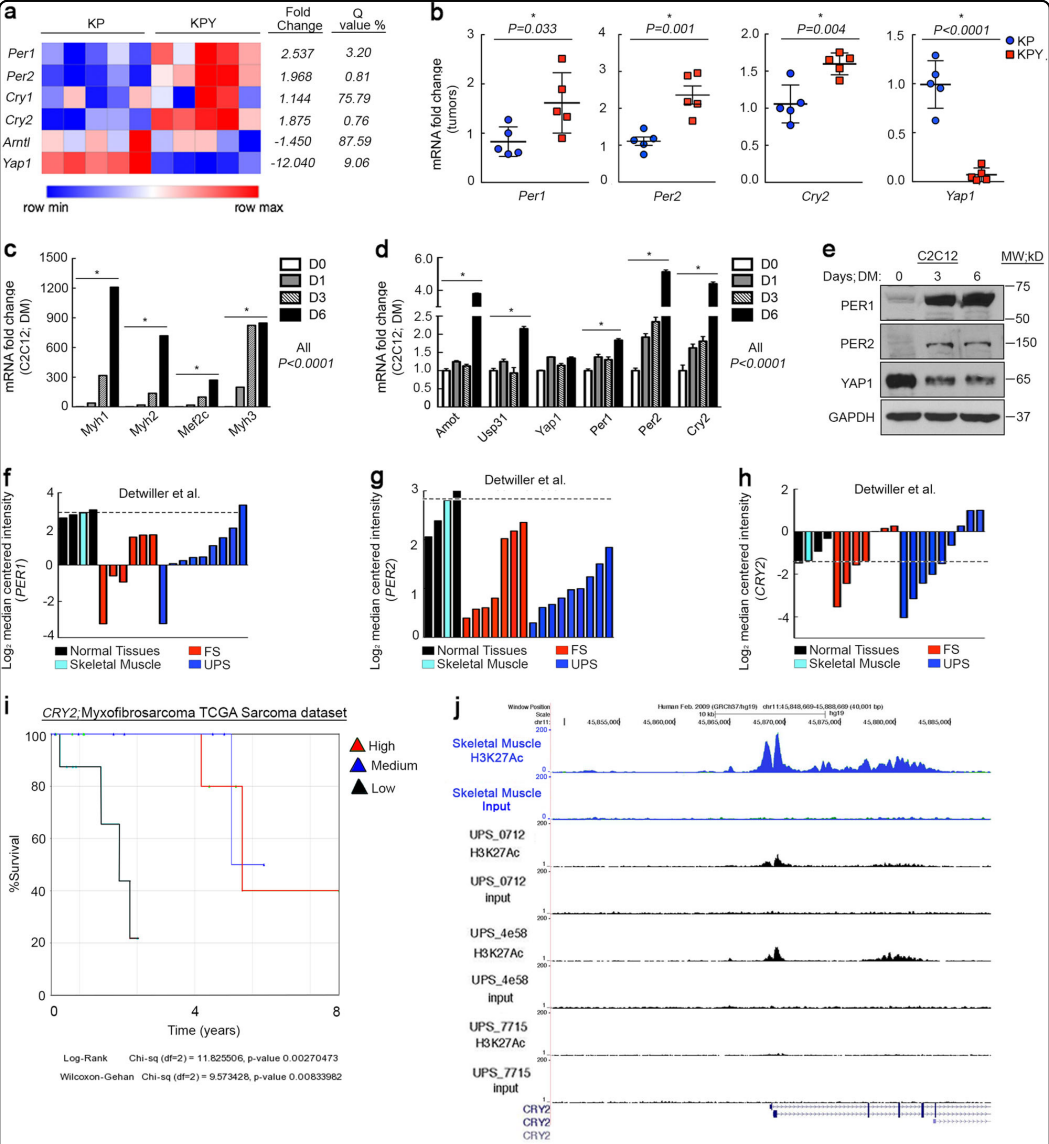
YAP1-dependent inhibition of circadian clock genes in UPS and proliferating myoblasts **a** Gene expression analysis of microarray performed on KP vs. KPY mouse tumors. **b** qRT-PCR validation of circadian clock gene expression in KP and KPY mouse tumors. **c** qRT-PCR of muscle differentiation genes and **d** Hippo/NF-κB**/**Circadian clock genes in proliferating (Day 0, D0) and differentiating (D1–D6) C2C12 myoblasts. **e** Western blot of Per1 and Per2 in C2C12 cells treated as in **d**. **f** Oncomine gene expression analysis of *PER1*, **g** *PER2*, and **h** *CRY2* in human tissues. **i** Kaplan–Meier survival curve of MFS patients in the TCGA sarcoma data set based on *CRY2* expression. **j** Gene tracks of H3K27ac ChIP-seq signal (rpm/bp) for H3K27ac at the *CRY2* locus in human skeletal muscle and human three independent human UPS samples. Error bars represent SD

We crossed *Yap1*^fl/fl^ and KP mice to generate *LSL*-*Kras*^G12D/+^; *Trp53*^fl/fl^; *Yap1*^fl/fl^ (KPY) animals and saw increased tumor latency and reduced tumor weight and volume, relative to KP^7^. To determine the functional role of Yap1 in UPS we performed microarray analysis of 5 KP/KPY tumors and observed differential expression of circadian clock genes (Fig. 1a), validated by qRT-PCR of RNA isolated from murine tumors (Fig. 1b). Circadian clock function is important for skeletal muscle mass maintenance and function^17,49^. In differentiating C2C12 murine myoblasts, we found increased expression of myogenesis-related targets (Fig. 1c), the Yap1 inhibitor *Amot*, clock genes, and the NF-κB-negative regulator, *Usp31* (Fig. 1d). We also observed enhanced Per1 and Per2 protein expression, which correlated with Yap1 loss (Fig. 1e). Therefore, we hypothesized that loss of clock gene expression results in de-differentiation and sarcomagenesis. Consistently, *PER1, PER2*, and *CRY2* are inhibited in human fibrosarcoma and UPS (Detwiller dataset) relative to normal skeletal muscle^50^ (Fig. 1f–h). Some fibrosarcomas, including myxofibrosarcomas, are now thought to be genetically indistinguishable from UPS^51^ and are therefore included in our studies. We investigated the relationship between long-term survival and clock gene expression and found that low levels of *CRY2* are associated with poor survival (Fig. 1i). To further assess *CRY2* expression we performed RNA-seq and ChIP-seq, using Histone 3-lysine 27 Acetylation (H3K27Ac) antibody in human skeletal muscle and multiple human UPS samples. *CRY2* expression is suppressed in UPS tumors compared with muscle^7^ and enrichment of H3K27Ac is lost in UPS at the *CRY2* locus (Fig.1j). H3K27Ac enrichment is associated with active transcription and potential enhancer activity. The *PER1* locus shows a similar pattern of H3K27Ac enrichment/gene expression (Supplementary Fig. S1a) but there was no statistically significant link between *PER1* and patient survival likely due to the relatively low number of samples.

### YAP1 suppresses circadian clock gene expression in UPS

**Fig. 2:**
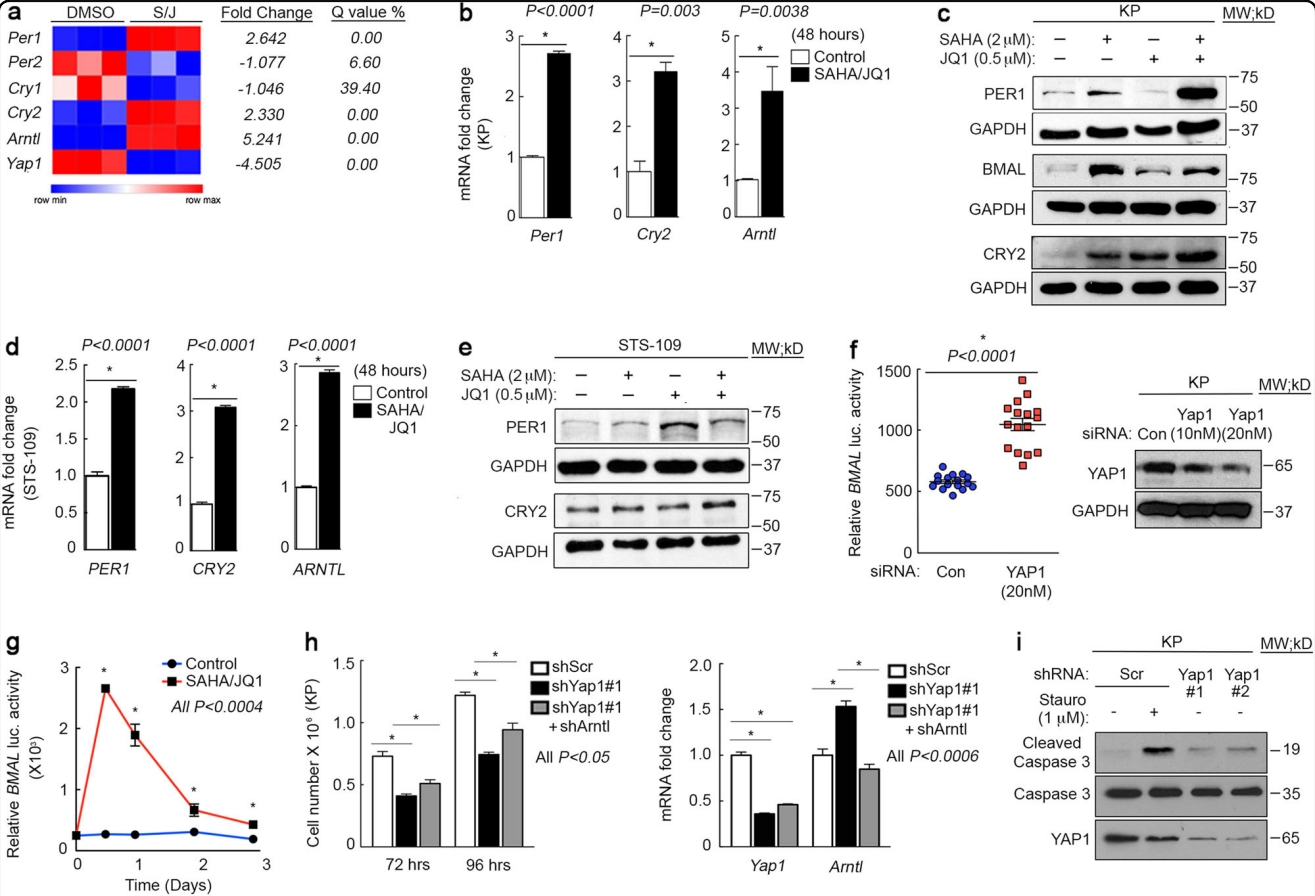
YAP1 enhances proliferation by suppressing the clock **a** Gene expression analysis of microarray performed on KP cells treated with 2 μm SAHA/0.5 μm JQ1 for 48 h. **b** qRT-PCR validation of clock genes in KP cells treated as in **a**. **c** Western blot of KP cells treated as in **a**. **d** qRT-PCR of human UPS cells (STS-109) treated as in **a**. **e** Western blot of STS-109 cells treated as in **a**. **f** (left) Bmal luciferase reporter assay in KP cells expressing *Yap1* siRNA (20 nM) and treated with 2 μm SAHA/0.5 μm JQ1 for 12 h to activate signaling. (right) Western blot of YAP1 levels in siRNA treated cells. **g** Bmal luciferase reporter assay in KP cells treated with 2 μm SAHA/0.5 μm JQ1. **h** (left) Cell counting rescue proliferation assay in *Yap1#1* and *Arntl* shRNA expressing KP cells. (right) qRT-PCR of *Yap1* and *Arntl* expression in KP cells treated as in **h**. **i** Western blot of KP cells expressing multiple  *Yap1* shRNAs and treated with 1 μm Staurosporin (12 h) as positive control. Error bars represent SD

We previously reported that YAP1 is downregulated in sarcoma cells treated with SAHA/JQ1 in vitro and in vivo and that ectopic expression of constitutively active YAP1 (YAPS6A) can rescue ~ 40% of proliferation loss associated with treatment^7^. We hypothesized that SAHA/JQ1 might reactivate clock gene expression owing to loss of YAP1 and observed upregulation of clock genes and proteins in treated mouse and human UPS cells (Fig. 2a–e), and HT-1080 human fibrosarcoma cells (Supplementary Fig. S1b). The subset of YAP1-dependent clock genes varied between in vivo and in vitro assays. However, YAP1 inhibition (shRNA) phenocopied SAHA/JQ1 treatment in KP cells, suggesting that any difference in target expression is due to experimental approach (Supplementary Fig. S1c). Lastly, PER1 and CRY2 antibodies were validated using siRNA (Supplementary Fig. S1d and e).

To determine whether YAP1 inhibition activates clock function we performed a Bmal luciferase reporter assay in KP cells using *Yap1*-specific siRNA, shRNA, and SAHA/JQ1. We observed increased Bmal reporter expression in all three systems, indicating that Bmal/circadian clock activity is YAP1-dependent (Fig. 2f, g and Supplementary Fig. S2a). To verify that clock reactivation suppresses proliferation in Yap1-depleted cells we performed an in vitro rescue assay using *Yap1*- and *Arntl*-specific shRNAs. We observed a ~ 40% proliferation rescue in double knockdown cells relative to cells expressing *Yap1* shRNA alone (black bar vs. gray bar) (Fig. 2h, left), which correlated with a ~ 40% reduction of *Arntl* expression in double knockdown cells relative to *Yap1 *shRNA alone (Fig. 2h, right and Supplementary Fig. S2b) (black bar vs. gray bar). This partial rescue suggested that other mechanisms are at play. Given that YAP1 can have anti-apoptotic effects^52,53^, we hypothesized that YAP1 loss initiated apoptosis in a significant portion of cells and therefore proliferation could not be rescued by clock suppression. Loss of Yap1 resulted in cell cycle arrest (Supplementary Fig. S2c) and increased caspase-3 cleavage in KP cells, indicating enhanced apoptosis (Fig. 2i). Thus, although our rescue phenotype may appear modest we conclude that we are rescuing proliferation in a significant fraction of non-apoptotic cells.

### Circadian clock genes are regulated by NF-κB downstream of YAP1

**Fig. 3:**
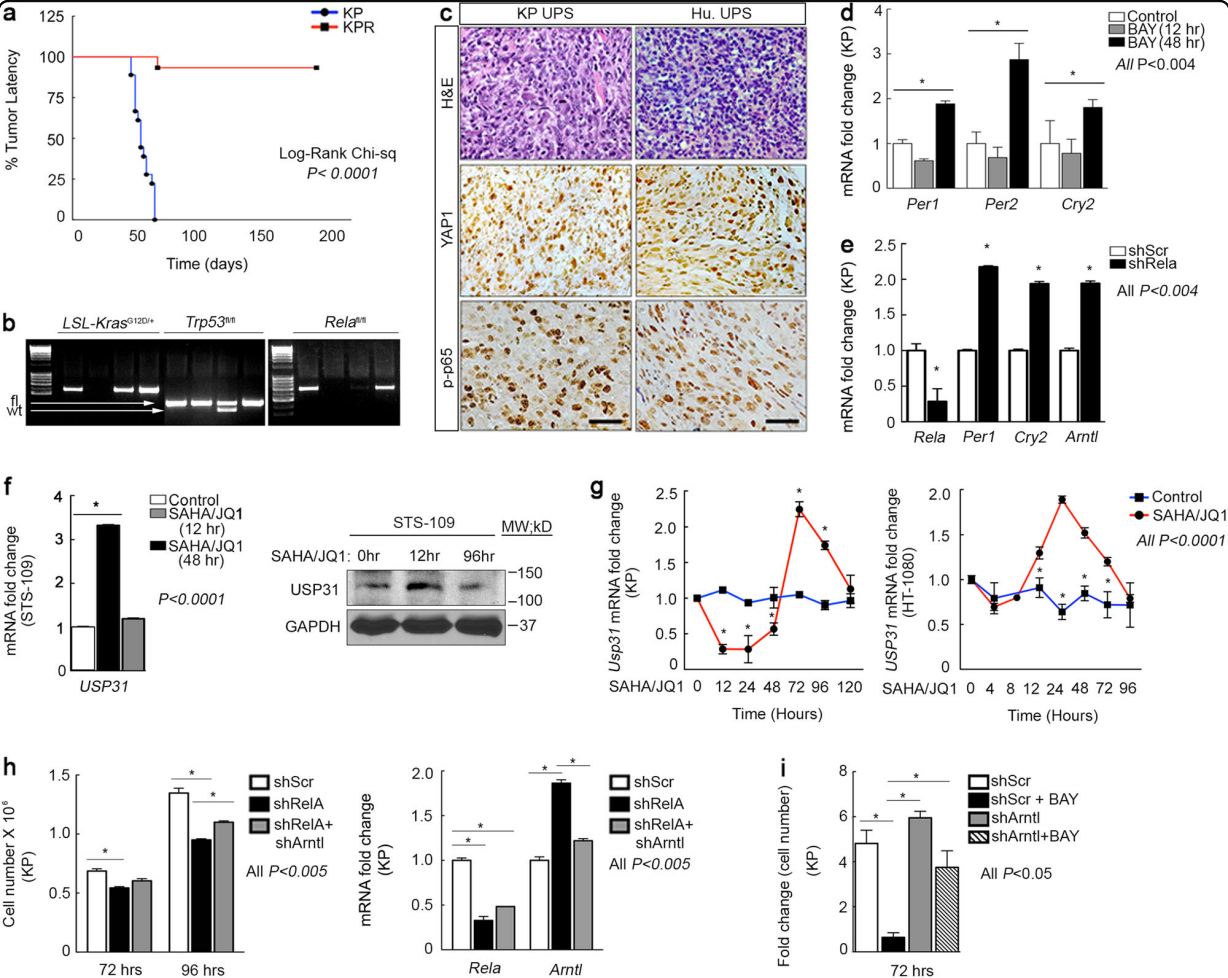
Inhibition of NF-κB, downstream of Yap1, restores clock gene expression **a** *Rela* deletion in the KP autochthonous model of UPS (*n* = 18 mice per group. Log-Rank Chi-sq. **b** Genotyping of KP and KPR mice. Kras band indicates the presence of the *Kras*^G12D^ mutant allele, p53 bands indicate wt and fl/fl alleles. RelA band indicates the presence of the fl/fl alleles. **c** IHC of KP and human xenograft UPS tumors. **d** qRT-PCR of KP cells treated with NF-κB inhibitor 1.5 μm BAY 11-7085 for 12, 48 h. **e** qRT-PCR of clock genes in KP cells expressing Rela shRNA. **f** (left) qRT-PCR and (right) western blot of *USP31* expression in 2 μm SAHA/0.5 μm JQ1-treated STS-109. **g** qRT-PCR of KP and HT-1080 cells treated with DMSO or 2 μm SAHA/0.5 μm JQ1 for 0–120 h. **h** (left) Cell counting rescue proliferation assay in *Rela* and *Arntl* shRNA expressing KP cells. (right) qRT-PCR of *Rela* and *Arntl* expression in KP cells treated as in left. **i** Cell counting rescue proliferation assay in *Arntl* shRNA expressing KP cells treated with 1.0 μm BAY 11-7085 for 72 h. Data are expressed as mean fold change and SEM relative to the cell numbers in samples treated for with DMSO or BAY for 24 h. Error bars represent SD **a**–**h**

YAP1 enhances UPS proliferation in part via upregulation of persistent NF-κB signaling^7^. Here, we interrogated the role of NF-κB in UPS and clock reactivation. We bred *Rela*^*fl/fl*^ mice into our KP model and found that genetic deletion of NF-κB, encoded by the *Rela* gene, prevents outgrowth of tumors (Fig. 3a, b). Phosphorylation of the p65 subunit of NF-κB indicates its transcriptional activation. Both KP tumors and subcutaneous xenografts of human UPS cells stain positively for YAP1 and phospho-p65 (Fig. 3c). We investigated whether YAP1 repression of clock genes is mediated by NF-κB and found that inhibition with BAY 11-7085 (1 µm) (Fig. 3d) or *Rela* shRNA increased clock gene expression (Fig. 3e). Importantly, NF-κB target expression oscillates over time in differentiating myoblasts^7^ and other cells^15^. YAP1 suppresses NF-κB oscillation, promoting persistently high activity, by controlling expression of USP31^7^. *Usp31* also oscillates in differentiating C2C12 cells (Fig. 1d) as well as human STS-109 (Fig. 3f), KP, and HT-1080 cells (Fig. 3g) treated with SAHA/JQ1 for 0–120 h. Interestingly, the period of oscillation varies with the cellular proliferation rate. Slower proliferation rates (KP > HT-1080 > STS-109) correlate with shorter time to maximal *USP31* induction by SAHA/JQ1. Ultimately *Usp31* is lost in KPY tumors relative to KP (Supplementary Fig. S3a and S3b). This finding is consistent with our Bmal luciferase reporter assay in which we observe that SAHA/JQ1 treatment initially induces clock activity followed by inhibition (Fig. 2g). Our findings suggest the provocative hypothesis that NF-κB oscillation may drive the circadian cycle in muscle and muscle-derived sarcoma subtypes. To verify that clock reactivation suppresses proliferation in NF-κB-depleted cells we performed an in vitro rescue assay using *Rela*- and *Arntl*-specific shRNAs (Fig. 3h, i). We observed ~ 20% rescue in proliferation (black bar vs. gray bar) (Fig. 3h, left), which correlates with reduction of *Arntl* in double knockdown cells (black bar vs. gray bar) (Fig. 3h, right). Similarly, BAY 11-7085 (1 µm) dramatically decreased KP cell proliferation, which was significantly rescued by *Arntl* inhibition (Fig. 3i). These data indicate that *Rela*-mediated suppression of the clock enhances proliferation.

### YAP1/NF-κB loss initiates a clock-mediated  UPR response

**Fig. 4:**
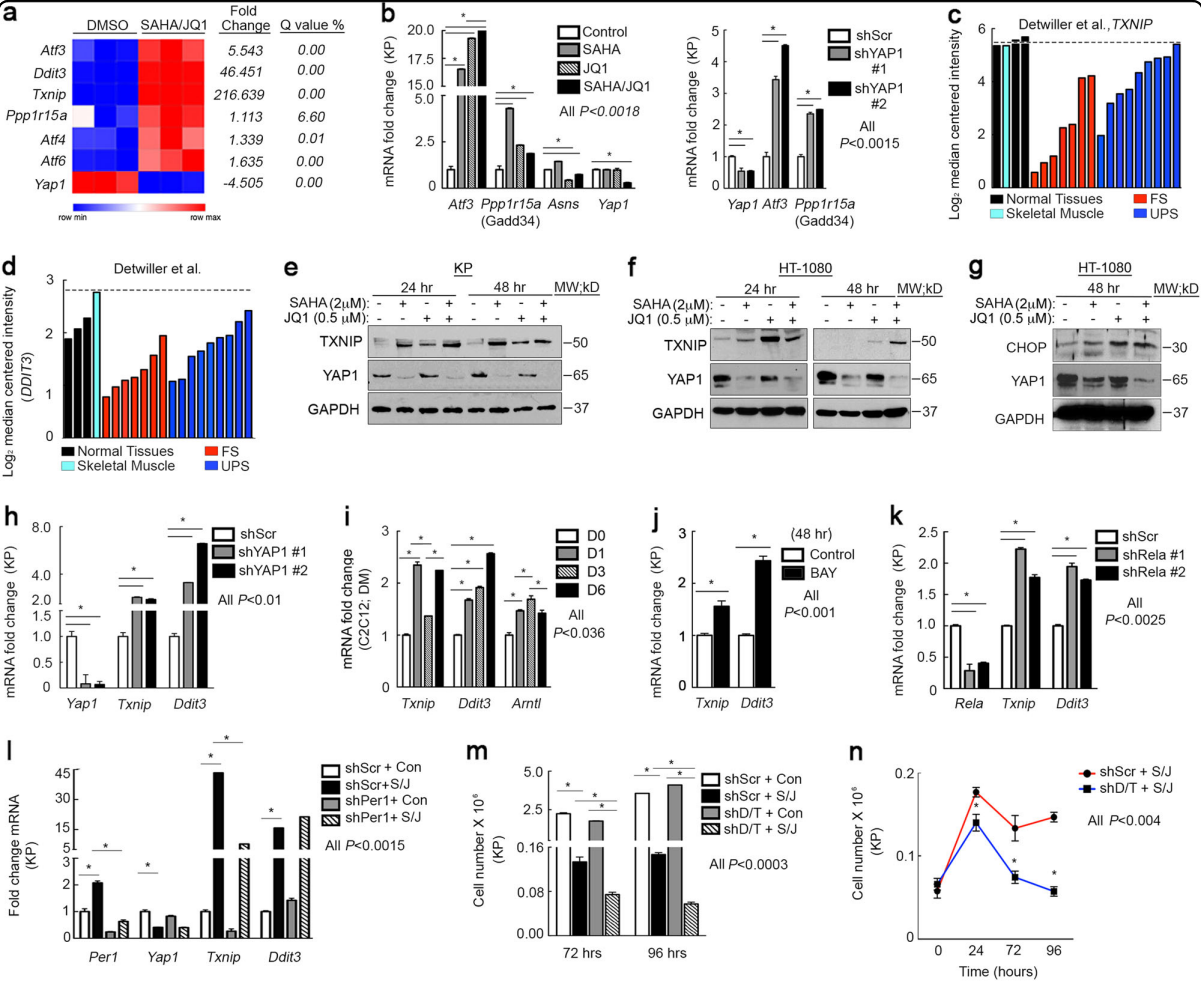
Inhibition of YAP1 and NF-kB activates UPR target expression necessary for survival **a** Gene expression analysis of microarray performed on KP cells treated with 2 μm SAHA/0.5 μm JQ1 for 48 h. **b** qRT-PCR validation of UPR genes in (left) KP cells treated as in **a** and (right) with multiple independent YAP1 shRNAs. **c** Oncomine gene expression analysis of *TXNIP* and **d** *DDIT3* in human tissues. **e** Western blot of TXNIP in KP and **f** HT-1080 cells treated as in **a**. **g** Western blot of CHOP in HT-1080 cells treated as in **a**. **h** qRT-PCR of *Txnip* and *Ddit3* in KP cells expressing two independent *Yap1* shRNAs. **i** qRT-PCR for *Txnip* and *Ddit3* genes in proliferating (Day 0, D0) and differentiating (D1–D6) C2C12 myoblasts. **j** qRT-PCR of KP cells treated with NF-κB inhibitor 1.5 μm BAY 11-7085 for 48 h. **k** qRT-PCR of *Txnip* and *Ddit3* in KP cells expressing *Rela* shRNAs. **l** qRT-PCR rescue assay of KP cells expressing *Per1* shRNA and treated with 2 μm SAHA/0.5 μm JQ1 for 48 h. **m** Cell counting rescue proliferation assay in cells expressing both *Txnip* and *Ddit3* shRNAs and treated as in **a**. D/T shRNAs denotes *Ddit3* and *Txnip* shRNAs were used for knockdown. **n** Cell counting proliferation assay of KP cells from **m** from 0 to 96 h. Error bars represent SD

Recent studies have established a link between the UPR and the circadian clock^29,54^. SAHA/JQ1 treatment upregulates multiple UPR target genes based on microarray analyses of KP cells (Fig. 4a). We validated induction of PERK and ATF6 pathways; two of three UPR branches, in SAHA/JQ1-treated cells (Fig. 4b, left) and cells expressing multiple independent Yap1 shRNAs (Fig. 4b, right). The main target of the third UPR branch, IRE1, is Xbp1. We did not observe *Xbp1* upregulation or splicing in treated cells (Supplementary Fig. S4a). The well-studied PERK and ATF6 targets, *Txnip* and *Ddit3/(*Chop*)*, were dramatically upregulated in SAHA/JQ1-treated KP cells (Fig. 4a). These targets are elevated in skeletal muscle but suppressed in fibrosarcomas and UPS^50^, suggesting that the UPR antagonizes sarcomagenesis in muscle-derived tumors (Fig. 4c, d). Consistent with these observations, TXNIP and CHOP protein expression increased in SAHA/JQ1-treated KP and HT-1080 cells (Fig. 4e–g) and KP cells expressing *Yap1*-specific shRNAs (Fig. 4h). To confirm upregulation of UPR in muscle, we evaluated *Txnip* and *Ddit3* in C2C12 myoblasts and observed increased expression in differentiated cells (Fig. 4i). To determine whether NF-κB mediates *Txnip* and *Ddit3* expression downstream of Yap1 we treated KP cells with BAY 11-7085 or *Rela* shRNAs and observed increases in *Txnip* and *Ddit3* expression (Fig. 4j, k). Next, we performed a rescue experiment to determine whether Yap1-mediated suppression of the circadian clock controls expression of *Txnip* and *Ddit3*. We treated KP cells with SAHA/JQ1 and *Per1* shRNA alone or in combination. Interestingly, *Txnip* induction is dependent on clock activity, whereas *Ddit3* expression is not (Fig. 4l). Importantly, this observation agrees with our evaluation of *Txnip* and *Ddit3* levels in differentiating C2C12 cells, wherein *Txnip* oscillates during a 6 day differentiation time course but *Ddit3*, simply increases at each time point (Fig. 4i). To ascertain the functional role of key UPR targets in SAHA/JQ1-induced differentiation we silenced *Txnip* and *Ddit3* with specific shRNAs. We observed that loss of *Txnip* increases the expression of *Ddit3* and the converse is true as well (Supplementary Fig. S4b). Therefore, we silenced both *Txnip* and *Ddit3* in SAHA/JQ1-treated cells to prevent compensation. Loss of *Txnip* and *Ddit3* in SAHA/JQ-treated cells alters the normally cytostatic effects of these inhibitors and results in cell death, suggesting that UPR supports survival in differentiating muscle and sarcoma cells (Fig. 4m, n). Thus, we conclude that the Yap1/NF-κB axis represses the PERK and ATF6 arms of the UPR, which are associated with clock activity and survival during differentiation^29,54–56^.

### SAHA/JQ1 treatment promotes oscillation of UPR target genes

**Fig. 5:**
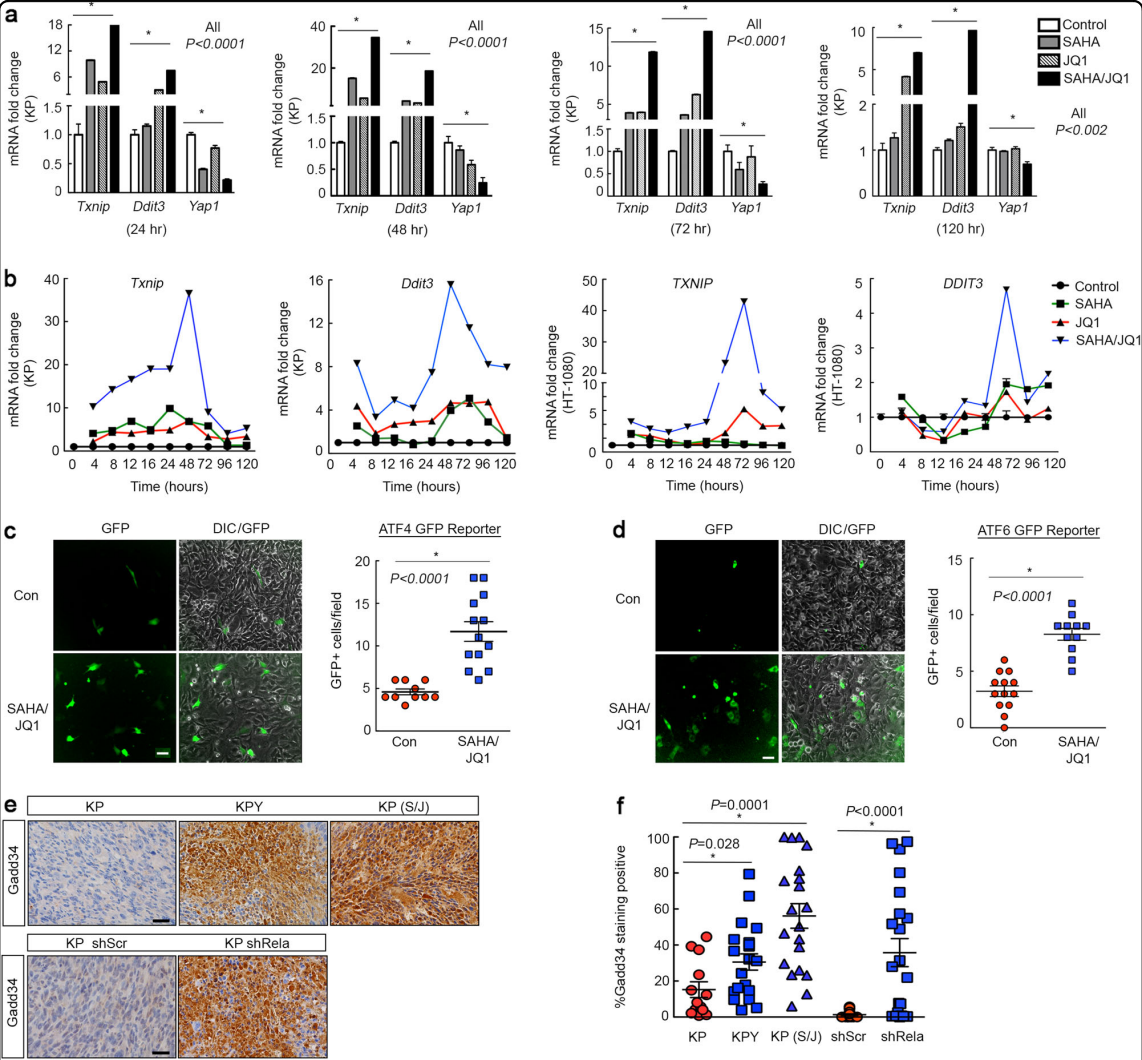
SAHA/JQ1 treatment promotes UPR target oscillation **a** qRT-PCR of KP cells treated with 2 μm SAHA and/or 0.5 μm JQ1 for 0–120 h. **b** Summary graphs of qRT-PCR for *TXNIP* and *DDIT3* treated as in **a**, **b** with the addition of 0–24 h time points. Some of the data summarized here are found in supplementary figure 5. **c** ATF4-GFP reporter assay in KP cells treated as in **a** for 24 h. Scale bar = 50 μm. **d** ATF6-GFP reporter assay in KP cells treated as in **a** for 24 h. Scale bar = 50 μm. **e** Scale bar = μm **f** Representative images of IHC from murine tumor sections from allograft experiments (shSCR, shRelA) and GEMMs (KP, KPY, and KP tumors treated with SAHA/JQ1 once daily with 25mg/kg SAHA and twice daily with 25 mg/kg JQ1). Tumors harvested after 20 days of treatment. Scale bar = 20 μm. **g** Quantification of Gadd34 expression from **f**. *n* = 3 mice per group, 12 images per tumor sample. Error bars represent SD

To explore the link between the UPR and circadian oscillation we characterized *TXNIP* and *DDIT3* in both SAHA/JQ1-treated UPS and fibrosarcoma cells. *TXNIP* mRNA expression exhibited a dramatic circadian-like oscillation pattern with high amplitude changes in KP and HT-1080 cells over 0–120 h of treatment, whereas the amplitude of *DDIT3* oscillations were more limited (Fig. 5a, b, Supplementary Fig. S5a and S5b). Oscillation was only observed in drug combination-treated cells, whereas individual drugs had minimal effects. Interestingly, YAP1 levels did not oscillate above 1 under these conditions, but instead decreased over time (Supplementary Fig. S5b). To test UPR engagement, in response to SAHA/JQ1, we treated KP cells expressing ATF4-GFP and ATF6-GFP reporters for 24 h and found substantial increases in GFP reporter activity (Fig. 5c, d). To demonstrate that YAP1 suppresses UPR in vivo we stained tumor sections from allografts expressing control (scr) and *Rela* shRNA as well as our KP, KPY, and SAHA/JQ1-treated KP tumors for the UPR target Gadd34 and found that it was upregulated in Yap1/NF-κB-deficient tumors (Fig. 5e, f). Next, we evaluated TXNIP and CHOP expression in human tissues and observed decreased nuclear staining in UPS relative to muscle (Supplementary Fig. S5c).

### Pharmacological and genetic inhibition of YAP1 alters sarcoma cell metabolism

**Fig. 6:**
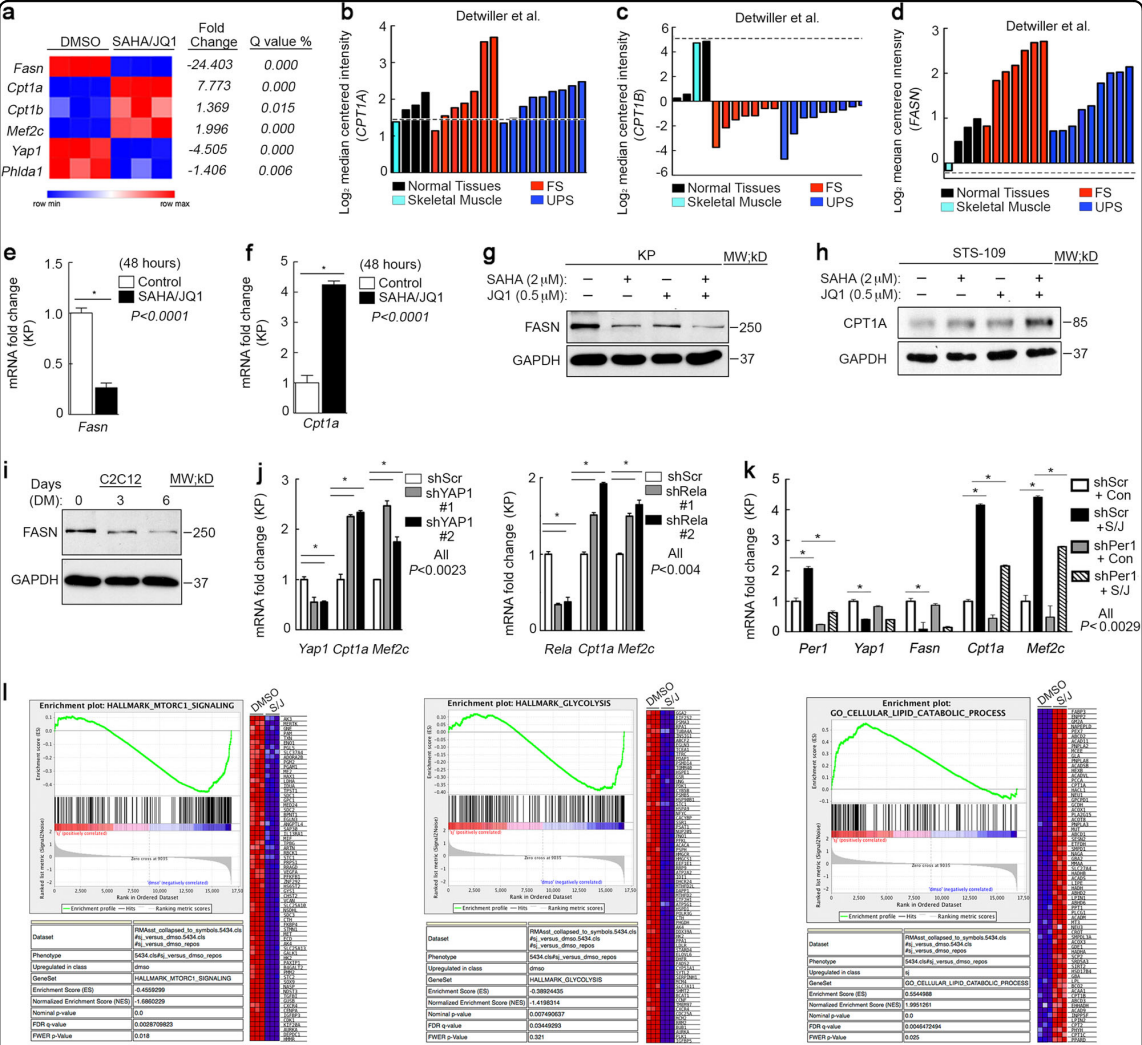
YAP1 loss alters sarcoma cell metabolism and initiates differentiation **a** Gene expression analysis of microarray performed on KP cells treated with 2 μm SAHA/0.5 μm JQ1 for 48 h. **b** Oncomine gene expression analysis of *CPT1A*, **c** *CPT1B*, and **d** *FASN* in human tissues. **e** qRT-PCR validation of *Fasn* and **f** *Cpt1a* in KP cells treated as in **a**. **g** Western blot of KP cells treated as in **a**. **h** Western blot of STS-109 cells treated as in **a**. **i** Western blot of proliferating (Day 0, D0) and differentiating (D1–D6) C2C12 myoblasts. **j** qRT-PCR of KP cells expressing (right) Yap1 shRNAs and (left) Rela shRNAs. **k** qRT-PCR rescue assay of KP cells expressing *Per1* shRNA and treated with 2 μm SAHA/0.5 μm JQ1 for 48 h. **l** GSEA analysis of microarray from KP cells treated with 2 μm SAHA/0.5 μm JQ1 for 48 h using the Broad Institute “hallmark” gene sets for “MTORC1 signaling”, “Glycolysis”, and “Lipid catabolic process”. Error bars represent SD

The primary known function of circadian circuitry is to regulate cellular metabolism^20,57^. Cancer cell metabolism is highly deregulated and favors the rapid, yet inefficient, energy production associated with glycolysis^57^. We hypothesized that reactivation of clock gene expression would alter the metabolic phenotype in sarcoma cells. Therefore, we evaluated key metabolic genes in our microarray of SAHA/JQ1-treated KP cells and observed differential expression of metabolism hallmarks, such as Fatty acid synthase (*Fasn)* and Carnitine Palmitoyltransferase 1a and b (*Cpt1a) (Cpt1b). CPT1A and CPT1B* are associated with β-oxidation of fatty acids, a slower but more efficient metabolic process, found in non-malignant cells^58^. CPT1B is the isoform generally associated with metabolism of differentiated muscle, whereas *CPT1A* can induce autophagy^59^. *Fasn* is associated with cancer metabolism and was downregulated dramatically in SAHA/JQ1-treated cells, whereas *Cpt1a and Cpt1b* and the muscle differentiation factor *Mef2c* were elevated (Fig. 6a). In human UPS and fibrosarcoma *CPT1A* is modestly elevated or unchanged relative to normal tissues (Fig. 6b), whereas muscle-specific *CPT1B* is lost and *FASN* is increased (Fig. 6c, d). Importantly, SAHA/JQ1 treatment restored a more muscle-like metabolic program by decreasing *Fasn* and upregulating *Cpt1a* in KP and human UPS cells (Fig. 6e–h). *Cpt1a* induction may compensate for *Cpt1b* in this context (Fig. 6a). Consistent with our prediction that metabolic changes are associated with differentiation, Fasn is substantially decreased in differentiated C2C12 cells (Fig. 6i). To determine whether these metabolic alterations were directly linked to the Yap1/NF-κB axis we inhibited *Yap1* (Fig. 6j, left) or *Rela* (Fig. 6j, right) in KP cells with shRNA and observed increased *Cpt1a* and *Mef2c* expression, suggesting that YAP1 is necessary to suppress differentiation, potentially via β-oxidation of fatty acids or autophagy mediated by the CPT1 enzymes. Next, we performed a rescue experiment to determine whether YAP1-mediated suppression of the circadian clock controls metabolism and differentiation. We treated KP cells with SAHA/JQ1 and *Per1* shRNA alone or in combination. We observed an increase in *Cpt1a* and *Mef2*c and loss of *Yap1* under SAHA/JQ1 treatment as predicted, and whereas Per1 loss under these conditions prevented induction of *Cpt1a* and *Mef2c* (Fig. 6k). We observed the same trends in KP cells treated with SAHA/JQ1 and *Arntl*-specific shRNA (Supplementary Fig. S6a). To further characterize the metabolic status of SAHA/JQ1-treated KP cells we performed GSEA of the DMSO vs. SAHA/JQ1 microarray and observed that “MTORC1 signaling” and “Glycolysis” are downregulated in treated cells (Fig. 6l), consistent with the observations of other groups^60,61^, whereas “lipid catabolic processes” are upregulated. Together, these data show that the Yap1/NF-κB axis promotes cancer-associated metabolism and that inhibition of this pathway allows expression of muscle markers and muscle-associated metabolism.

### YAP1, but not NF-κB, suppresses autophagy in UPS cells

**Fig. 7:**
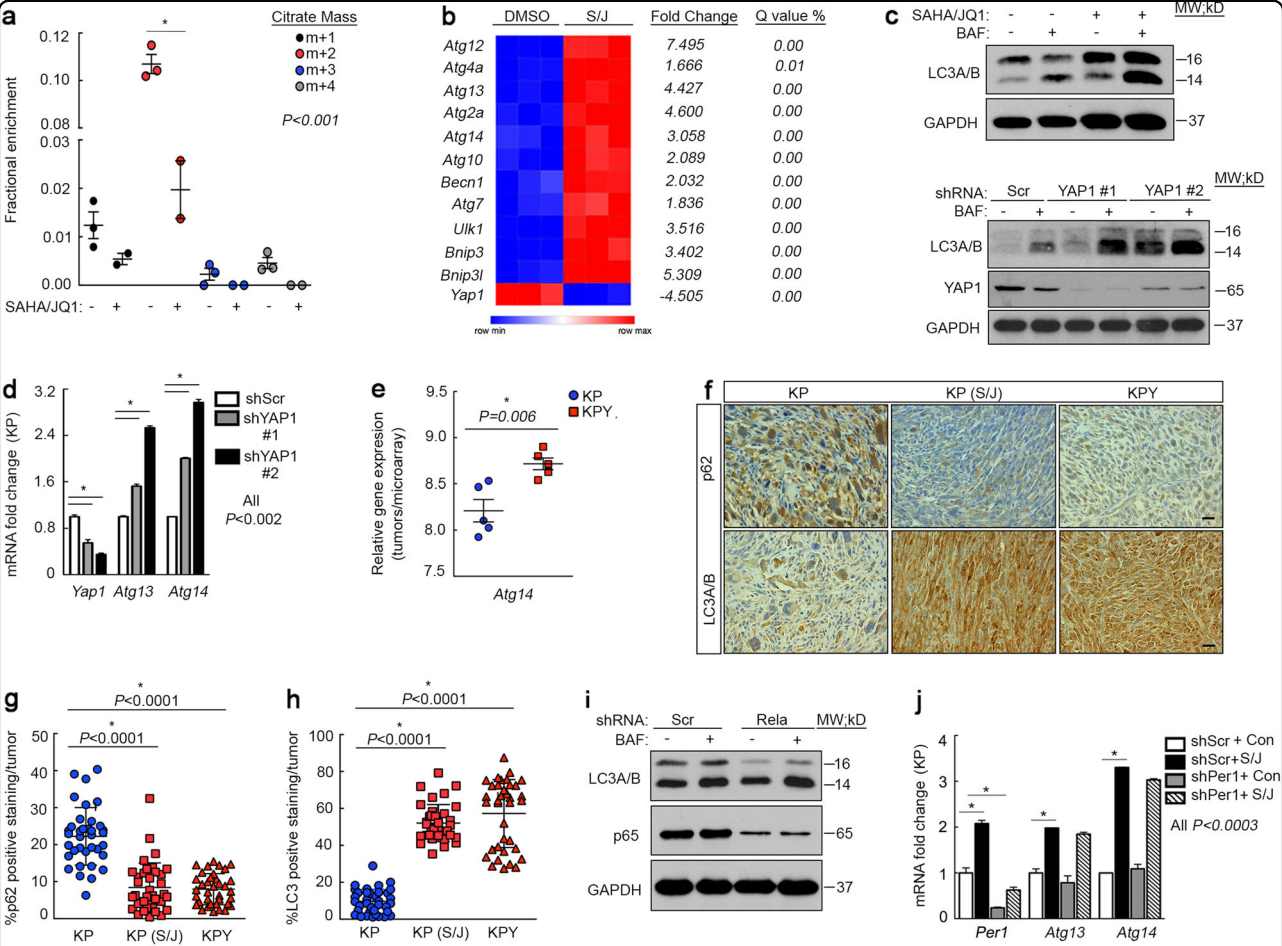
YAP1 suppresses autophagy in sarcoma cells independent of NF-kB **a** GC/MS of KP cells treated with 2 μM SAHA/0.5 μM JQ1 for 48 h. **b** Gene expression analysis of microarray described in **a**. **c** (top) Western blot of KP cells treated as in A with the addition of BAF during the last 6 h of treatment. (bottom) Western blot of YAP1 shRNA expressing cells treated with BAF as in the top panel. **d** qRT-PCR of *Atg13* and *Atg14* in KP cells expressing *Yap1* shRNA. **e**qRT-PCR of KP and KPY tumors. **f** Representative images of IHC from murine tumor sections from KP, KPY, and KP tumors treated with SAHA/JQ1 once daily with 25mg/kg SAHA and twice daily with 25mg/kg JQ1. Tumors harvested after 20 days of treatment. Scale bar = 20 μm. **g** Quantification of p62 expression from **f**. *n* = 3 mice per group, 12 images per tumor sample. **h** Quantification of LC3B expression from **f** *n* = 3 mice per group, 12 images per tumor sample. **i** Western blot of KP cells expressing Scr or Rela shRNAs treated as in **a** with the addition of BAF during the last 6 h of treatment. **j** qRT-PCR rescue assay of KP cells expressing *Per1* shRNA and treated with 2 μM SAHA/0.5 μM JQ1 for 48 h. Error bars represent SD

**Fig. 8:**
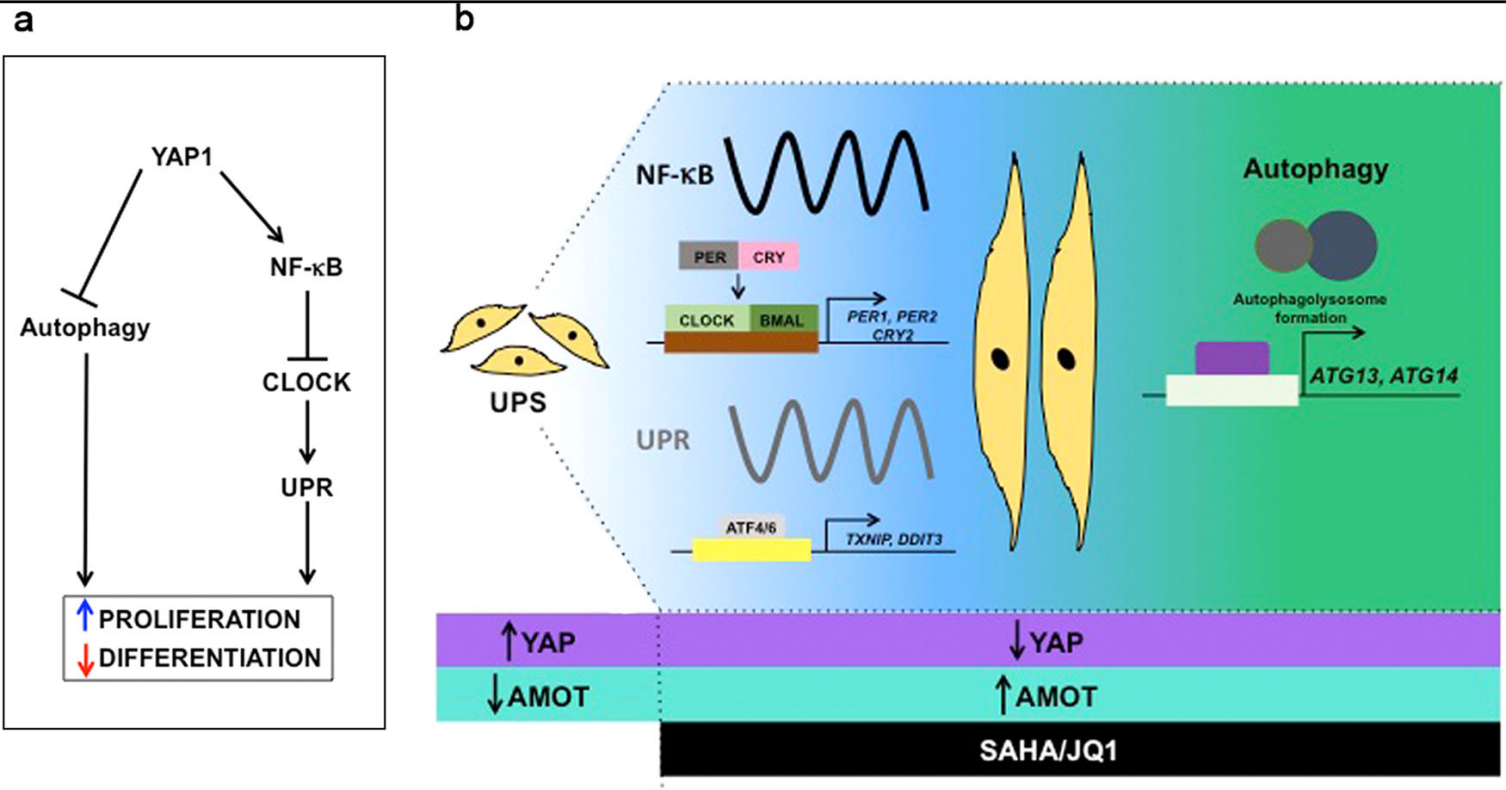
Model of YAP1/NF-κB-mediated clock control Model of YAP1/NF-κB-mediated clock control of UPR and NF-κB-independent control of autophagy

In addition to its established role in fatty-acid oxidation, CPT1A is also associated with upregulation of autophagy^59^. To determine which of these two processes occurs during SAHA/JQ1-mediated differentiation we performed GCMS evaluating oxidation of 75 μM [U-^13^C_16_]palmitic acid and 75 μM [U-^13^C_18_]oleic acid via enrichment of TCA cycle intermediates and observed a reproducible decrease in β-oxidation of fatty acids in SAHA/JQ1-treated cells (Fig. 7a). These data suggest that increased expression of CPT1A may enhance oxidation of endogenous rather than exogenous lipids. Therefore, we investigated the ability of this drug combination to induce autophagy. Autophagy is directly associated with muscle function and maintenance of muscle mass^32,34^. The DMSO vs. SAHA/JQ1 microarray revealed that treatment upregulates many genes associated with autophagy including *Atg13* and *Atg14*, which are specifically linked to muscle development and function (Fig. 7b). We also saw dramatic induction of the autophagy marker LC3 in KP cells treated with SAHA/JQ1 and Bafilomycin (BAF) (Fig. 7c, top). BAF interrupts autophagic flux by inhibiting maturation of autophagic vacuoles during the late stages of autophagy, forcing accumulation of the autophagosome-associated LC3-II. Consistently, shRNA-mediated depletion of Yap1 enhanced LC3A/B expression in BAF-treated cells (Fig. 7c, bottom) and upregulated *Atg13* and *Atg14* expression (Fig. 7d). *Atg14* expression was also increased in KPY relative to KP tumors (Fig. 7e). Most significantly, we observed loss of p62 and dramatic accumulation of LC3B in vivo in KPY tumors and KP tumors treated with SAHA/JQ1 (Fig. 7f-h). SAHA/JQ1 treatment was initiated when KP tumors reached 100 mm^3^ according to the schedule found in (Supplementary Fig. S6b). Together, these findings link YAP1 to autophagy regulation in UPS. At last, we investigated whether YAP1-mediated suppression of autophagy was NF-κB- and clock-dependent. *Rela* inhibition had no effect on LC3A/B accumulation (Fig. 7i) or *Atg13* and *Atg14* levels (Supplementary Fig. S6c). Moreover, loss of Per1 in SAHA/JQ1-treated KP cells revealed that clock modulation has no effect on *Atg13* and *Atg14* (Fig. 7j) and that *Atg13* does not oscillate at all during C2C12 differentiation, whereas Atg14 oscillates extremely modestly (Supplementary Fig. S6d). These data indicate that autophagy is likely not required for muscle differentiation but is required for muscle maintenance and function as demonstrated by other groups^34,62^. At last, we investigated potential cross-talk between the UPR and autophagy. We treated KP cells expressing *Txnip/Ddit3*-specific shRNAs with SAHA/JQ1 and BAF. *Txnip/Ddit3* inhibition had no effect on SAHA/JQ1-mediated autophagy (Supplementary Fig. S6e). We conclude from these observations that YAP1 suppresses autophagy independent of NF-κB signaling (Fig. 8a, b).

## Discussion

Though well studied in epithelial tumors, the specific downstream effectors of YAP1 in sarcomas are still being elucidated. Characterization of these effectors is necessary for the development of effective biomarkers and targeted therapies to treat YAP1-dependent tumors including UPS. Previously, we showed that YAP1 promotes proliferation via persistent elevated NF-κB signaling^7^. The goal of this study was to elucidate the mechanisms by which YAP1 and NF-κB impact muscle de-differentiation and promote tumorigenesis. Here we show that YAP1-mediated NF-κB signaling represses UPR and circadian clock activity, both of which are required for muscle differentiation^17,56^. We also show that YAP1 inhibits autophagy in an NF-κB-independent manner.

To our knowledge, this work provides the first direct link between the Hippo pathway and circadian clock activity. Using our autochthonous mouse models, we showed that Yap1-mediated NF-κB activity disrupts normal circadian oscillation by suppressing *Per1, Per2*, and *Cry2* levels. Consistent with this observation, *PER1, PER2*, and *CRY2* are downregulated in human UPS^7^. We also demonstrate that genetic or pharmacological inhibition of YAP1 enhances circadian clock activity and the oscillation of key targets including *Txnip*, which is a major effector of the UPR^63,64^.

One critical purpose of the UPR and circadian clock is regulation of metabolic processes^55^. We found that Yap1-mediated suppression of the UPR and clock support a shift in metabolism toward cancer cell-associated glycolysis and hyper-proliferation. Reactivation of the UPR and clock via SAHA/JQ1 correlate with decreased glycolysis while enhancing autophagy and lipid catabolism, thus promoting skeletal muscle differentiation.

Our work sheds light on several on-going areas of cancer research. First, our observation that the clock and the UPR are both activated in differentiating myoblasts, as well as SAHA/JQ1-treated sarcoma cells, indicates that the relationship between these two processes is context-dependent. In some tissues the UPR antagonizes clock gene expression^29^, whereas in skeletal muscle and muscle-derived tumors both pathways are critical for differentiation and can be simultaneously upregulated^17,56,65^. This idea is consistent with several studies showing that UPR is necessary for muscle regeneration and maintenance of muscle mass^55,56,65,66^.

 Clock function is also necessary for muscle generation and differentiation. In fact, MyoD, is a direct transcriptional target of the molecular clock^17,67,68^. Moreover, clock-deficient mice suffer from muscle weakness, cachexia, and disrupted metabolism^65^. However, little is known about upstream signaling inputs that control clock gene expression and function. Now we appreciate that aberrant YAP1 stabilization impacts these processes in muscle-derived sarcomas and potentially other contexts as well. In future studies, we will determine how YAP1 suppresses clock, UPR gene expression, and autophagy. YAP1 is generally considered a transcriptional activator. As such, our findings highlight novel roles for YAP1 in suppressing transcription. The targets repressed by YAP1 are particularly intriguing to us. Whereas, we might predict that YAP1 would inhibit expression of pro-apoptotic genes our work suggests that YAP1, via NF-κB, also represses pro-differentiation genes. This hypothesis is supported by data indicating that Yap1 inhibits muscle differentiation in C2C12 myoblasts^10^.

 Finally we sought to determine the utility of epigenetic modulation in the treatment of muscle-derived sarcomas. We previously reported their impressive efficacy in the KP GEMM^7^. Here we validate our earlier finding that this strategy inhibits proliferation and enhances differentiation. We have identified key differentiation targets including clock, UPR, and autophagy genes as biomarkers of SAHA/JQ1 efficacy and suggest that this therapeutic strategy and these markers may offer clinical benefit to some sarcoma patients.

## Electronic supplementary material

Sup Figure 1

Supp Figure 2

Supp Figure 3

Supp Figure 4

Supp Figure 5

Supp Figure 6

Supplementary figure legends
