# Supplementary figures and images for: YAP1 enhances NF-κB-dependent and independent effects on clock-mediated unfolded protein responses and autophagy in sarcoma
Source: Cell Death Dis. 2018 Oct 31;9(11):1108. doi: 10.1038/s41419-018-1142-4 (PMC6208433; doi:10.1038/s41419-018-1142-4)

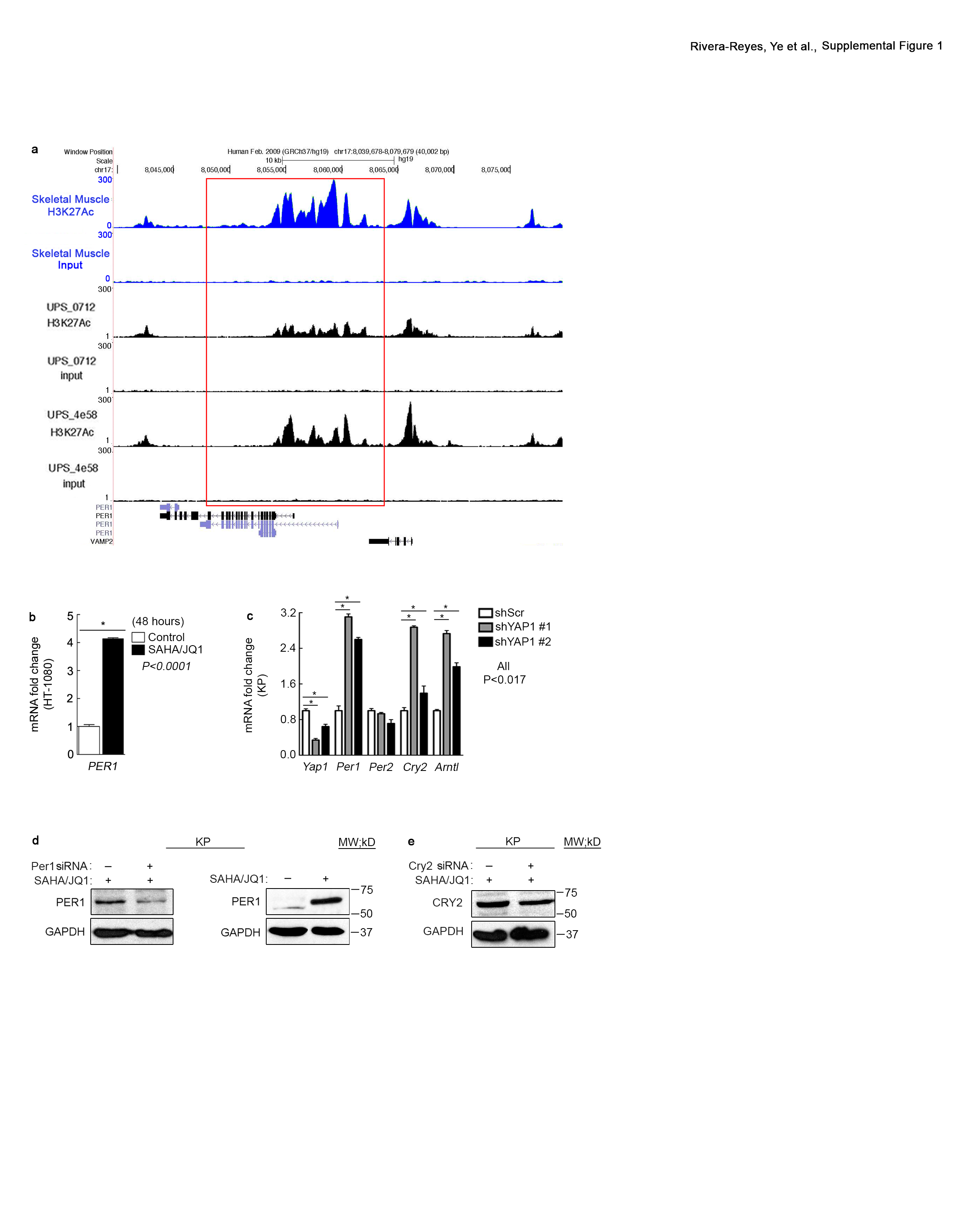

Supplement: Supplementary file 1 — Sup Figure 1 [file 41419_2018_1142_MOESM1_ESM.tif]

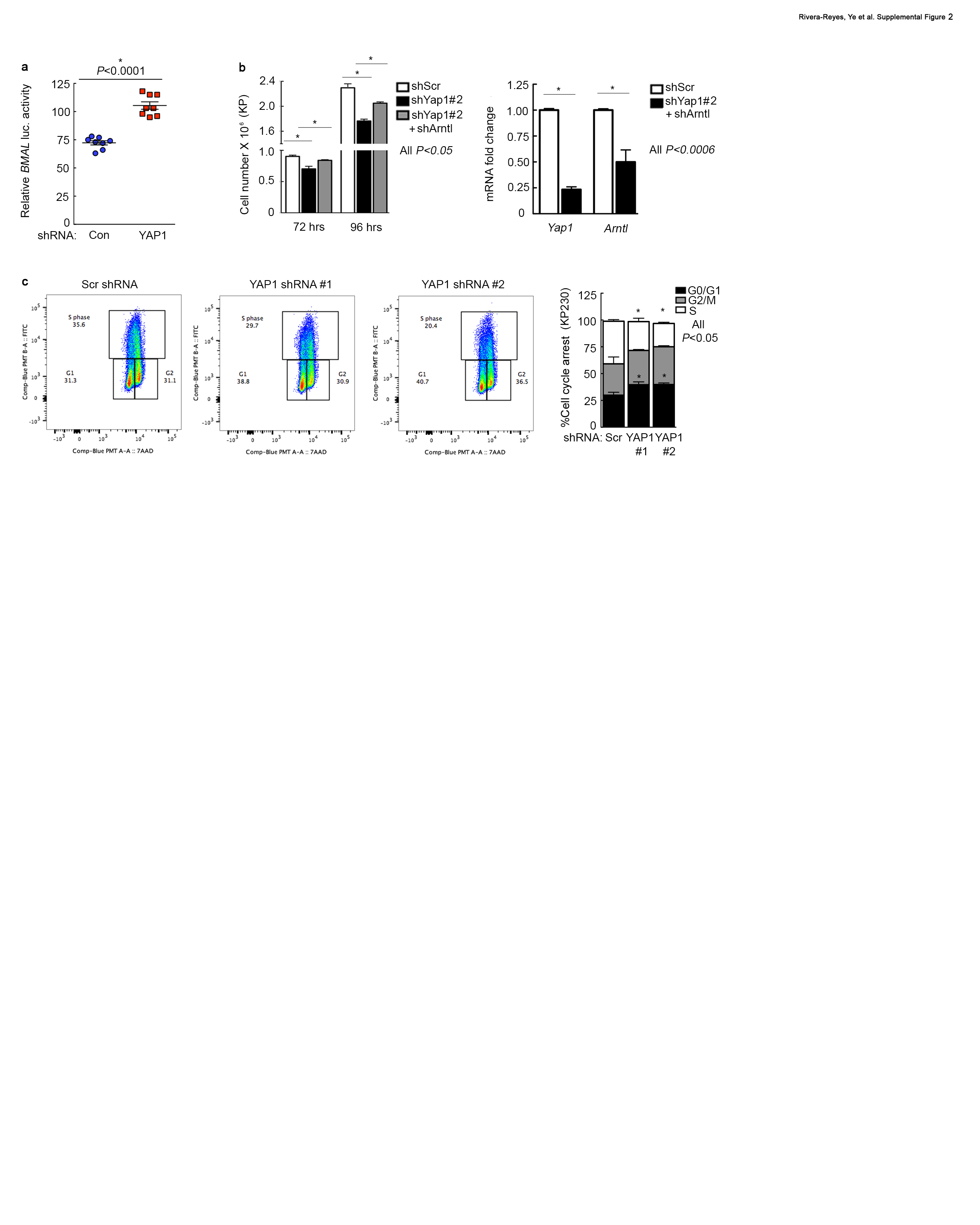

Supplement: Supplementary file 2 — Supp Figure 2 [file 41419_2018_1142_MOESM2_ESM.tif]

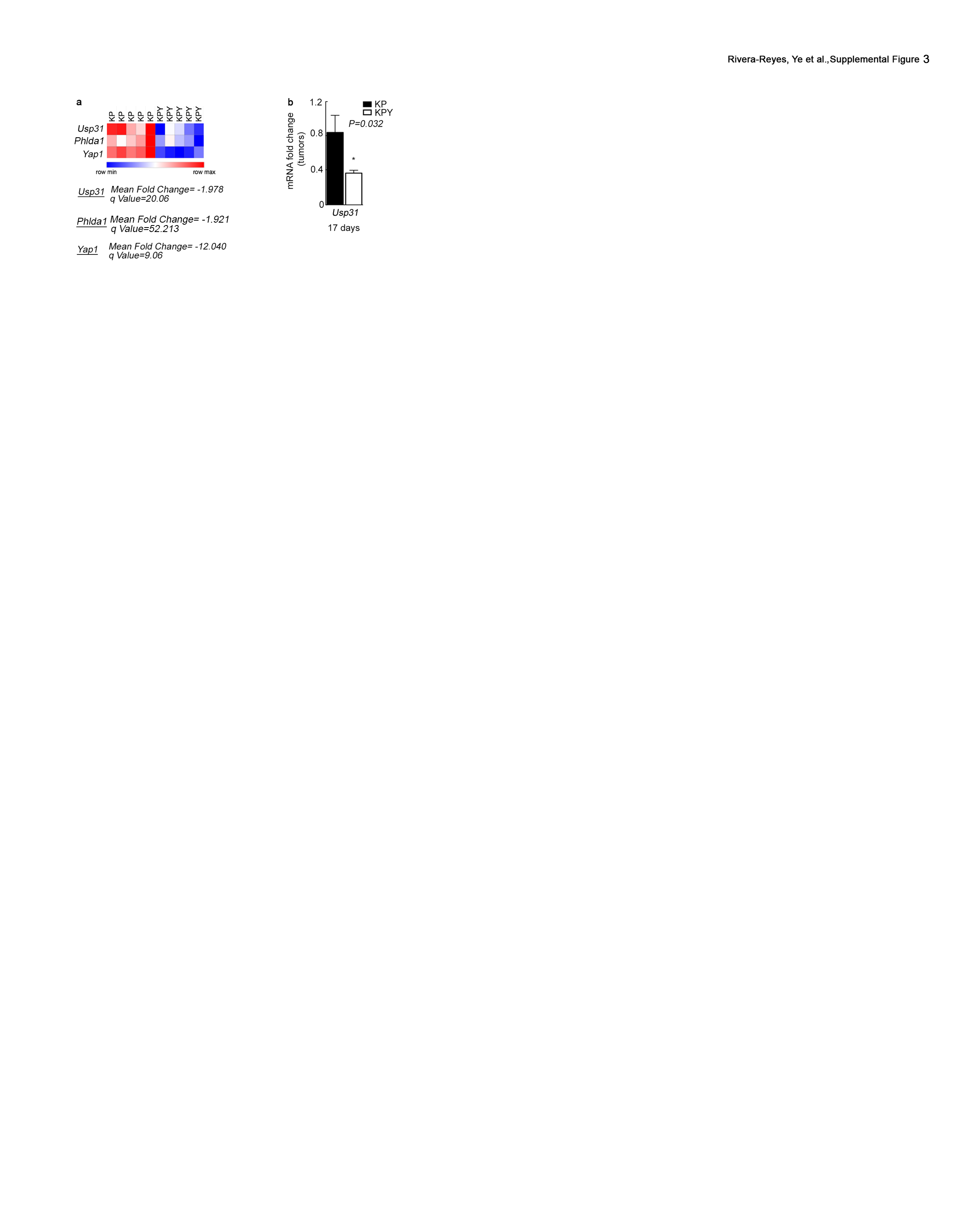

Supplement: Supplementary file 3 — Supp Figure 3 [file 41419_2018_1142_MOESM3_ESM.tif]

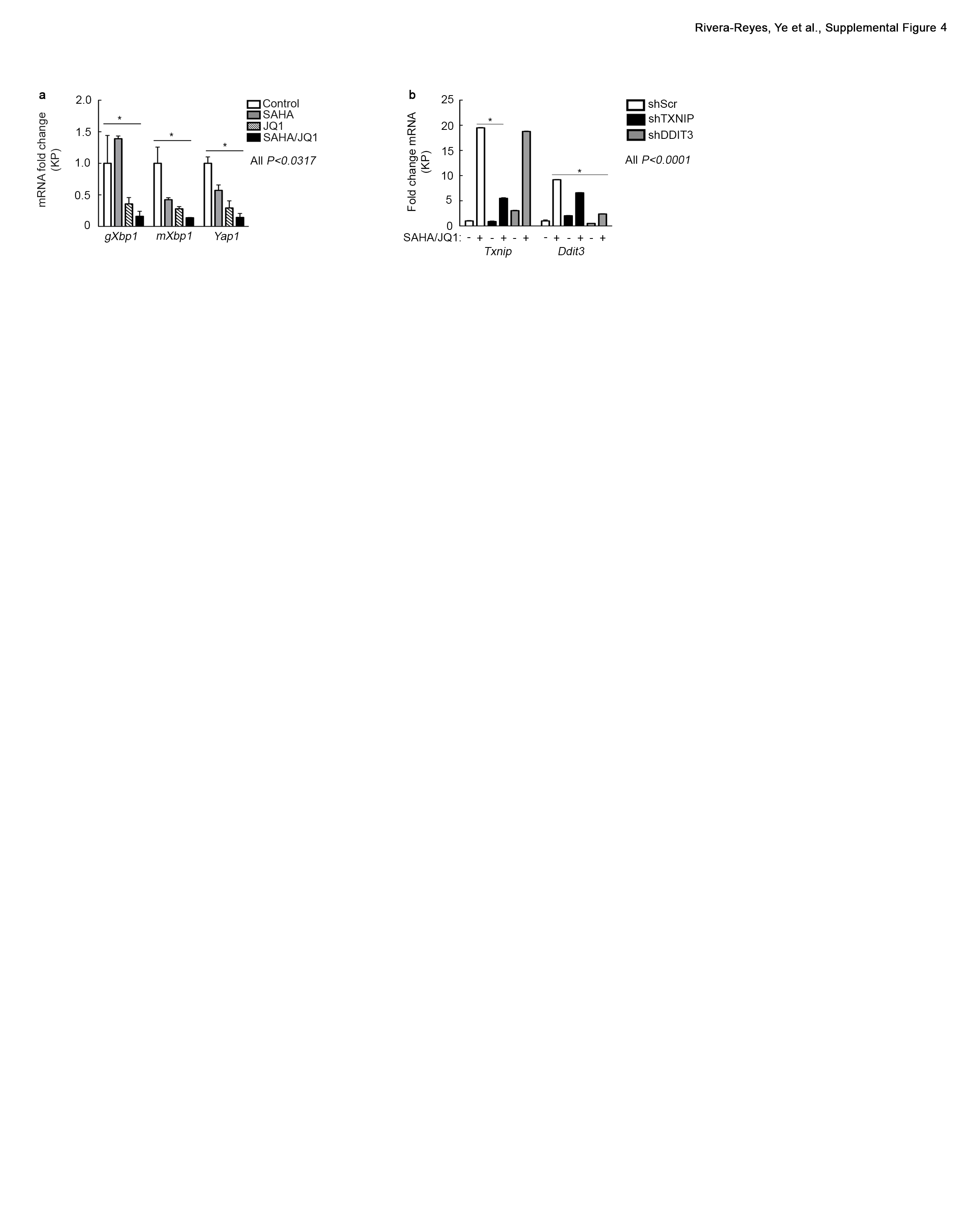

Supplement: Supplementary file 4 — Supp Figure 4 [file 41419_2018_1142_MOESM4_ESM.tif]

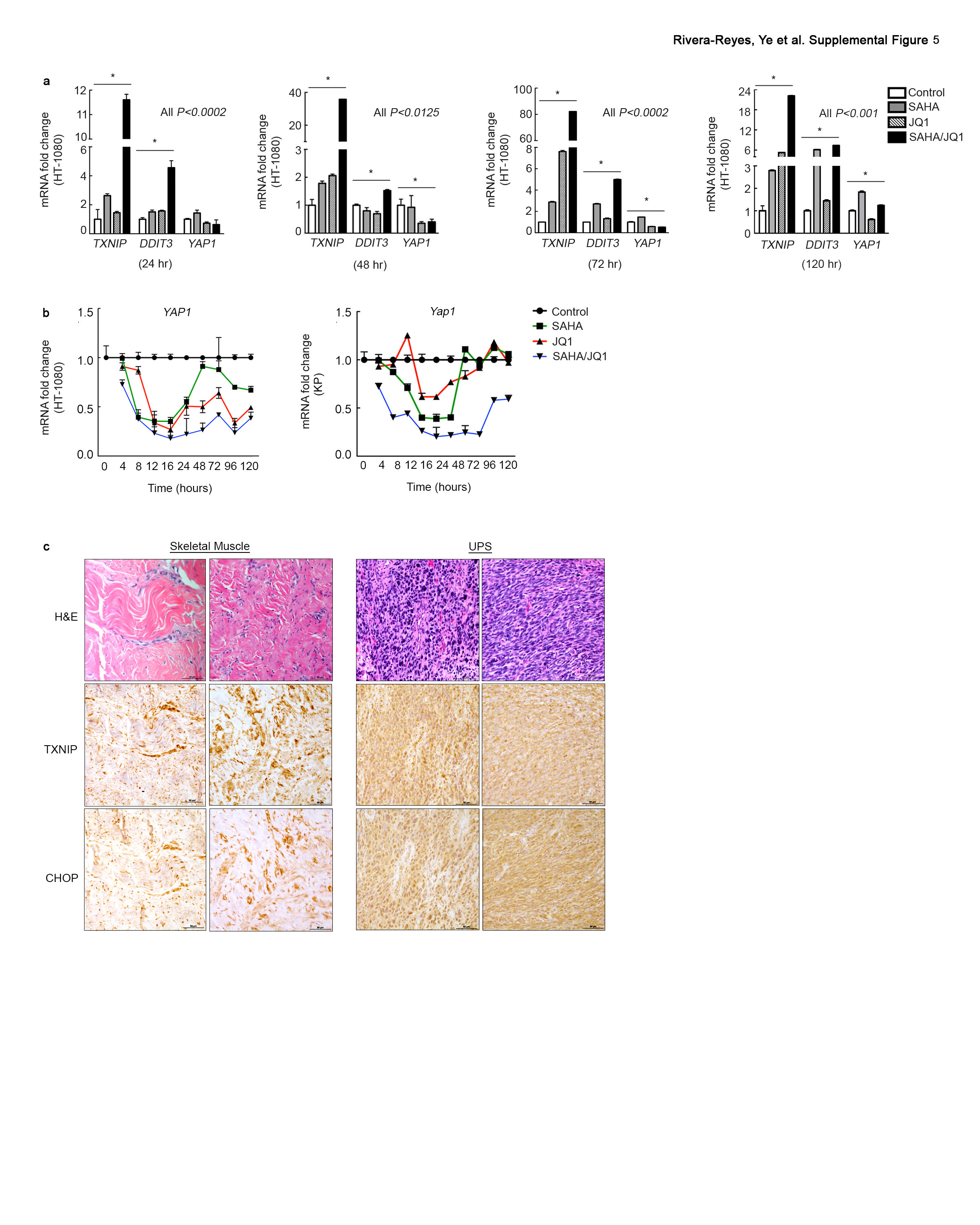

Supplement: Supplementary file 5 — Supp Figure 5 [file 41419_2018_1142_MOESM5_ESM.tif]

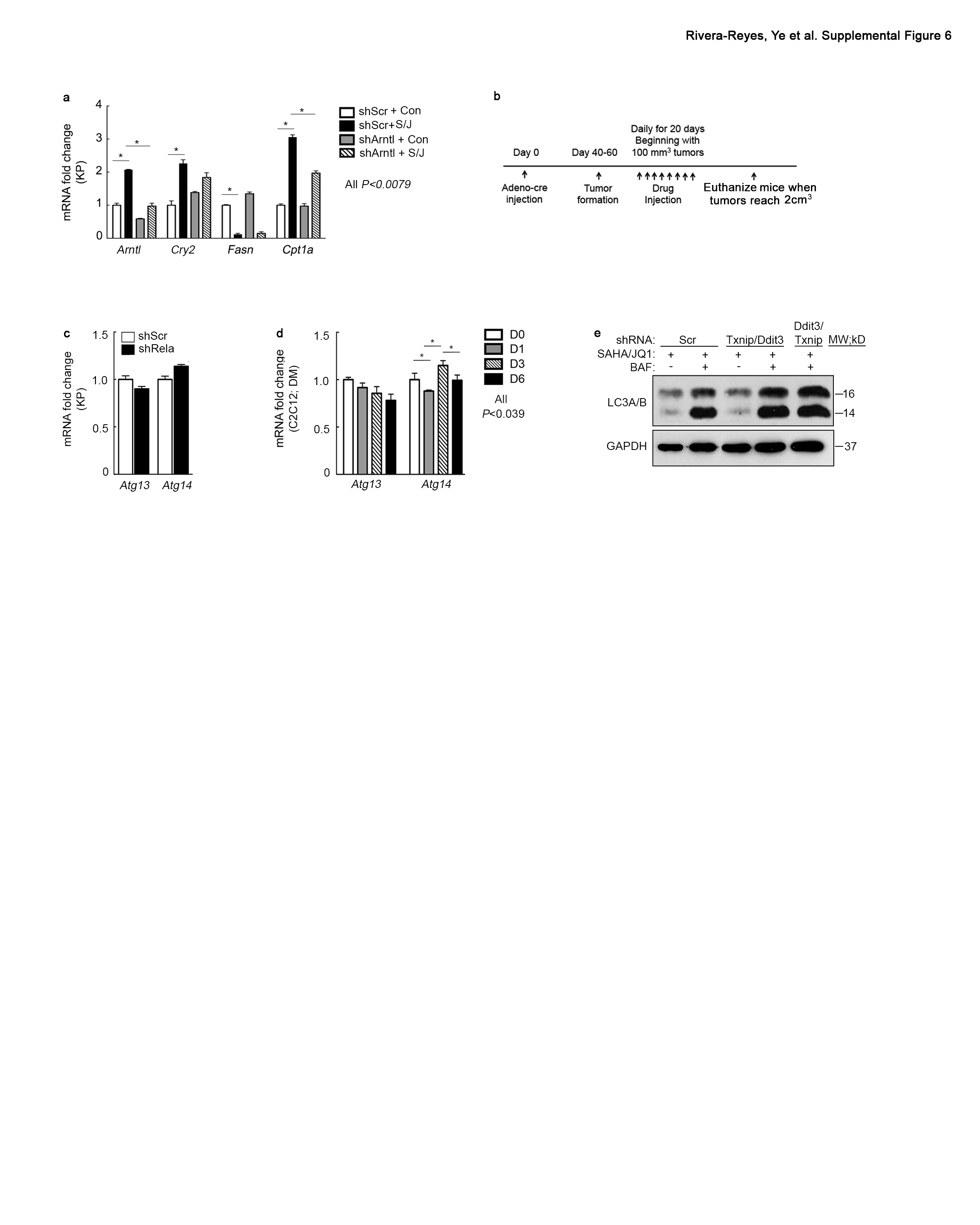

Supplement: Supplementary file 6 — Supp Figure 6 [file 41419_2018_1142_MOESM6_ESM.tif]
