# Supplementary material for: YAP1 enhances NF-κB-dependent and independent effects on clock-mediated unfolded protein responses and autophagy in sarcoma
Source: Cell Death Dis. 2018 Oct 31;9(11):1108. doi: 10.1038/s41419-018-1142-4 (PMC6208433; doi:10.1038/s41419-018-1142-4)
Supplement: Supplementary file 7 — Supplementary figure legends [file 41419_2018_1142_MOESM7_ESM.docx]

**Supplementary Fig. S1. Clock genes in human UPS and muscle.** A) Gene tracks of H3K27ac ChIP-seq signal (rpm/bp) for H3K27ac at the *PER1* locus in human skeletal muscle and human 3 independent human UPS samples. B) qRT-PCR of HT-1080 cells treated with 2 μM SAHA/ 0.5 μM JQ1 for 48 hrs. C) qRT-PCR of KP cells expressing YAP1 shRNA. D) Western blot of KP cells treated as in B with or without PER1 siRNA to identify the correct band. E) Western blot of KP cells treated as in B with or without Cry2 siRNA to identify the correct band.

**Supplementary Fig. S2. Loss of YAP1 causes modest cell cycle arrest in UPS.** A) Bmal luciferase reporter assay in KP cells expressing *Yap1* shRNA. B) (left) Cell counting rescue proliferation assay in *Yap1#2* and *Arntl* shRNA expressing KP cells. (right) qRT-PCR of *Yap1* and *Arntl* expression in KP cells treated as in H. C) Representative flow cytometry plots and quantitation of BrdU incorporation in HT-1080 cells expressing multiple independent YAP1 shRNAs. Error bars represent SD.

**Supplementary Fig. S3. *Usp31* expression in KP and KPY tumors.** A) Gene expression analysis of microarray performed on KP vs. KPY mouse tumors. The NF-κB target, *Phlda1*, is included as a control. B) qRT-PCR validation of *Usp31* gene expression in KP and KPY mouse tumors. Error bars represent SD.

**Supplementary Fig. S4. SAHA/JQ1 activation of UPR is IRE1-independent.** A) qRT-PCR of KP cells treated with 2 μM SAHA/ 0.5 μM JQ1 for 48 hrs. B) qRT-PCR of KP cells expressing *Txnip* or *Ddit3* shRNAs treated with 2 μM SAHA/ 0.5 μM JQ1 for 48 hrs. Error bars represent SD.

**Supplementary Fig. S5. YAP1 levels in SAHA/JQ1-treated sarcoma cells** A) qRT-PCR of HT-1080 cells treated with 2 μM SAHA and/or 0.5 μM JQ1. B) qRT-PCR of HT-1080 (left) and KP cells (right) treated with 2 μM SAHA/ 0.5 μM JQ1 for 0-120 hrs. C) Representative IHC of normal TXNIP and CHOP expression in human skeletal muscle and UPS tissues. Scale bar= 50 μm.

**Supplementary Fig. S6. Autophagy is independent of NF-kB and UPR in UPS.** A) qRT-PCR of KP cells expressing Arntl shRNA and treated with 2 μM SAHA/ 0.5 μM JQ1 for 48 hrs. B) Drug scheduling for KP GEMM treatment beginning when tumors measure 100mm^3^. C) qRT-PCR of KP cells expressing Rela shRNA. D) qRT-PCR for *Atg13* and *Atg14* genes in proliferating (Day 0, D0) and differentiating (D1-D6) C2C12 myoblasts. E) Western blot of KP cells expressing both *Txnip* and *Ddit3* shRNAs and treated as in B with the addition of BAF during the last 6 hrs of treatment. *Txnip/Ddit3* denotes that KP cells were first transfected with *Txnip* shRNA followed by *Ddit3* shRNA, and inverse for *Ddit3/Txnip* lane. Error bars represent SD.
